# Surface chemistry governs the sub-organ transfer, clearance and toxicity of functional gold nanoparticles in the liver and kidney

**DOI:** 10.1186/s12951-020-00599-1

**Published:** 2020-03-14

**Authors:** Xue Li, Bing Wang, Shuang Zhou, Wei Chen, Hanqing Chen, Shanshan Liang, Lingna Zheng, Hongyang Yu, Runxuan Chu, Meng Wang, Zhifang Chai, Weiyue Feng

**Affiliations:** 1 CAS Key Laboratory for Biomedical Effects of Nanomaterials and Nanosafety, Institute of High Energy Physics, Chinese Academy of Sciences, Beijing, 100049 China; 2 University of Chinese Academy of Sciences, Beijing, 100049 China; 3 Institute of Health Sciences, Anhui University, Hefei, 230601 Anhui China; 4 School of Environmental and Material Engineering, Yantai University, Beijing, 264005 China; 5 State Key Laboratory of Radiation Medicine and Protection, School for Radiological and Interdisciplinary Sciences (RAD-X), Soochow University, Suzhou, 215123 Jiangsu China

**Keywords:** Surface chemistry, Gold nanoparticles, Sub-organ transfer, Clearance

## Abstract

**Background:**

To effectively applied nanomaterials (NMs) in medicine, one of the top priorities is to address a better understanding of the possible sub-organ transfer, clearance routes, and potential toxicity of the NMs in the liver and kidney.

**Results:**

Here we explored how the surface chemistry of polyethylene glycol (PEG), chitosan (CS), and polyethylenimine (PEI) capped gold nanoparticles (GNPs) governs their sub-organ biodistribution, transfer, and clearance profiles in the liver and kidney after intravenous injection in mice. The PEG-GNPs maintained dispersion properties in vivo, facilitating passage through the liver sinusoidal endothelium and Disse space, and were captured by hepatocytes and eliminated via the hepatobiliary route. While, the agglomeration/aggregation of CS-GNPs and PEI-GNPs in hepatic Kupffer and endothelial cells led to their long-term accumulation, impeding their elimination. The gene microarray analysis shows that the accumulation of CS-GNPs and PEI-GNPs in the liver induced obvious down-regulation of *Cyp4a* or *Cyp2b* related genes, suggesting CS-GNP and PEI-GNP treatment impacted metabolic processes, while the PEI-GNP treatment is related with immune responses.

**Conclusions:**

This study demonstrates that manipulation of nanoparticle surface chemistry can help NPs selectively access distinct cell types and elimination pathways, which help to clinical potential of non-biodegradable NPs.

## Background

The emerging interdisciplinary field of nanomedicine combines nanotechnology with pharmaceutical and biomedical sciences with the goals of developing higher efficacy drugs, drug delivery systems, therapeutics, and imaging agents, while also improving safety and toxicological profiles [1]. Gold nanoparticles (GNPs) have unique optical and thermal characteristics along with tunable size, shape, and surface chemistry, and thus have been used in a wide range of biomedical applications including drug delivery, biomedical imaging, diagnosis, cancer treatment, and others [2]. However, despite their interesting properties and range of developing technologies, there are only a few examples of GNP clinical trials approved by the US Food and Drug Administration [3]. The main challenge lies in the lack of deep understanding about the interactions of nanomaterials (NMs) with off-target organs, mainly liver and kidney, which are major accumulation sites as well as vital organs in the metabolism and clearance of NMs. To effectively use NMs in medicine, one of the top priorities is to address a better understanding of the possible sub-organ transfer, clearance routes, and potential toxicity of the NMs during these processes in the liver and kidney [4, 5].

The size, shape, and surface chemistry of NMs are key factors that determine their performance in vivo, including their interaction with biological molecules, cellular uptake, transfer, and excretion patterns [5–8]. Particles < 10 nm have been shown to be cleared via the kidney [9]; while larger particles (> 10 nm) are often rapidly sequestered from the blood where they accumulate and are cleared through the liver and mononuclear phagocyte system (MPS) [5]. The shapes of NMs have been shown to dictate endocytosis by either normal or cancer cells, as well as clearance by the MPS [10, 11]. Surface chemistry has been shown to particularly influence NM cellular uptake, protein “corona” formation, and subsequent immune system activation, as well as biodistribution [12, 13]. Importantly, to effectively use NMs in biomedicine, the appropriate surface modifications are necessary in order for them to resist rapid clearance, pass-through biological barriers, and rapidly distribute to target organs and tissues [14]. For example, GNPs can be modified to have surface functional groups containing thiols, phosphines, and amines that enhance their aqueous dispersibility, biocompatibility, and targeting efficacy, form gold nanoconjugates, and anchor additional moieties such as oligonucleotides, proteins, and antibodies for targeted effects [15, 16]. In drug-delivery and imaging applications, the hydrophilic moiety polyethylene glycol (PEG) is widely used as functional agent on the surface of GNPs to prolong blood circulation time after intravenous injection by reducing absorption of serum proteins and hindering uptake by macrophages [2, 17]. Chitosan (CS) is a biodegradable, biocompatible, nontoxic, hydrophilic, linear polysaccharide polymer that has been widely used in drug delivery, gene therapy, and tissue engineering [18, 19]. CS-capped GNPs have been developed as carriers for insulin delivery [20] and small hairpin RNA delivery [21], as antibacterial agents [22], and for tumor targeting [23]. Polyethylenimine (PEI)-capped GNPs more efficiently delivered plasmid DNA to cells than the commonly used PEI (60 kDa) [24]. It should be noted that for these active and passive targeted nanoparticle (NP) applications, the NPs will be typically structural distributions, with the potential implications for biological controls. It is important to assess the diversity of NP functions; however, few studies have reported the effects of functional groups on in vivo hepatic and renal transfer and clearance of NPs.

In this study, we investigated how surface chemistry governs sub-organ transfer, and clearance of PEG-, CS- and PEI-capped GNP nanoconjugations after intravenous (i.v.) injection in mice. Our results showed that the surface chemistry of GNPs plays a critical role in particle dispersion and stability in vivo*,* which determine their cellular uptake, endothelial transfer within organs, and final excretion. We further evaluated the stability of GNPs and adsorption of serum proteins in physiological environment to reveal the mechanisms underlying functional GNP interactions in vivo. We performed gene expression profiling in the liver after GNP delivery to identify nanosurface chemistry sensitive genes during GNP bioaccumulation.

## Results

### Functional GNPs

**Fig. 1:**
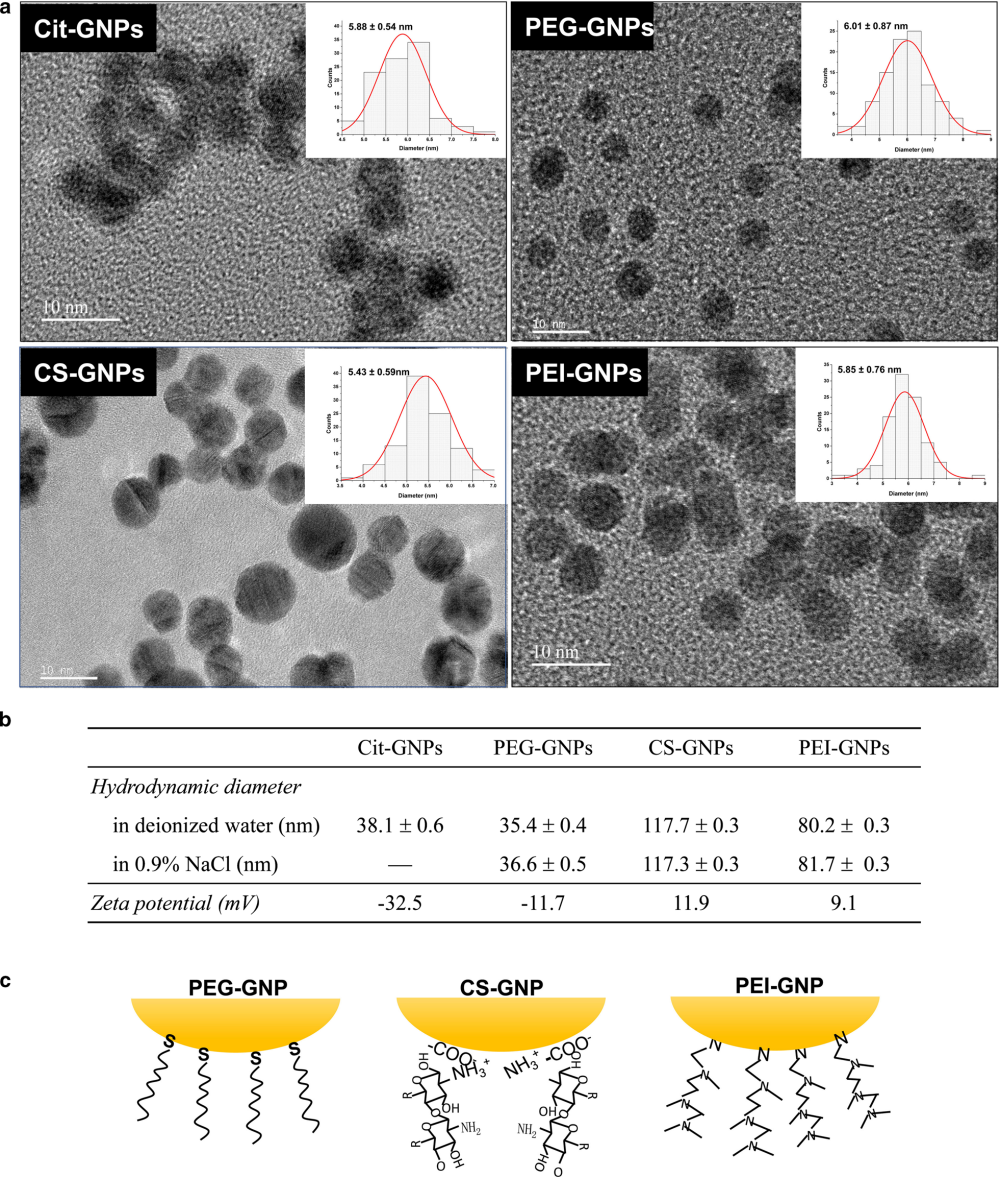
Physicochemical characterization of functional GNPs. **a** TEM images of functional GNPs. The scale bar represents 10 nm. **b** Hydrodynamic diameter and zeta potential measurements of GNPs. **c** Structure and binding modes of ligands on the surface of GNPs

To explore how surface chemistry impacts the bio-distribution, transfer, excretion, and biological effects of GNPs in vivo, we designed three types of functional GNPs: PEG-GNPs, CS-GNPs, and PEI-GNPs. The schematic procedures for synthesis of citrate coated GNPs (Cit-GNPs), PEG-GNPs, CS-GNPs and PEI-GNPs are shown in Additional file 1: Figure S1. TEM imaging (Fig. 1a) showed that the three functional GNPs were uniform in size (~ 6 nm) and in morphology (spherical), similar to Cit-GNPs. Dynamic light scattering measurements indicated that the hydrodynamic diameter of PEG-GNPs in water was similar to Cit-GNPs, while much larger hydrodynamic diameters were obtained for CS-GNPs and PEI-GNPs in either water or saline solution, suggesting that the particles become agglomerated (Fig. 1b). Zeta-potential detection showed that Cit- and PEG-GNPs had negative surface charge; however, PEG-GNPs had much less negative charge than non-PEGylated particles. The CS-GNPs and PEI-GNPs both had positive surface charge (Fig. 1b). The FTIR and XPS spectra showed that the PEG, CS, and PEI ligands had successfully capped on GNPs and presented different binding modes (Additional file 1: Figures S2, S3 and Table S1). PEG-SH and PEI ligands displaced with Cit ligands on GNPs surface, while CS ligand crosslinked with Cit ligands via ionic interactions (Fig. 1c). Detailed data analysis about binding modes of chemical ligands to GNPs was given in additional file.

### Stability of ligand-coated GNPs in a physiological environment

Surface chemistry may influence protein adsorption and determine biological fate in vivo; therefore, we evaluated the stability and adsorption of serum proteins for each type of ligand-coated GNP. UV–Vis spectra analysis reflects GNP size and aggregation in physiological conditions. The localized surface plasmon resonance (LSPR) bands of PEG-GNPs and CS-GNPs displayed no differences in saline or in bovine serum protein (BSA), immunoglobulin G (IgG), and transferrin (Tf) solutions (Additional file 1: Figure S4), suggesting that these GNPs have good dispersion stability in a physiological environment. Furthermore, the zeta potential of PEG-GNPs in BSA, IgG, or Tf solutions was similar to that in deionized water, with only a small increase in hydrodynamic diameter (Additional file 1: Figure S5A), suggesting that the PEG-GNPs reduce serum protein adsorption, as demonstrated in previous work [25]. While, the zeta potentials of positively charged CS-GNPs were significantly changed in the serum protein solutions, in some cases even becoming negatively charged, suggesting that CS-GNPs tend to adsorb serum proteins. In contrast, the LSPR bands of Cit-GNPs and PEI-GNPs showed an obvious red shift and widening, indicating that agglomeration/aggregation had occurred. Particularly, the presence of protein (BSA, IgG, and Tf) obviously improved agglomeration/aggregation state of Cit-GNPs. The obvious agglomeration/aggregation of PEI-GNPs, particularly in IgG and Tf solutions, indicating that protein driven aggregation of PEI-GNPs [26]. Agarose gel electrophoresis results were consistent with the zeta potential measurements. The PEG-GNPs moved down the gel very little both before and after incubation with BSA, indicating an electrically neutral surface and little BSA absorption (Additional file 1: Figure S5B & C). The CS-GNPs, which had adsorbed BSA, moved toward the positive electrode. No motility was observed with the PEI-GNPs because of their significant aggregation in BSA solution (Additional file 1: Figure S5B, C). The wide band observed toward the negative electrode may indicate detachment of some PEI from the GNP surface.

### Blood kinetics of functional GNPs

**Fig. 2:**
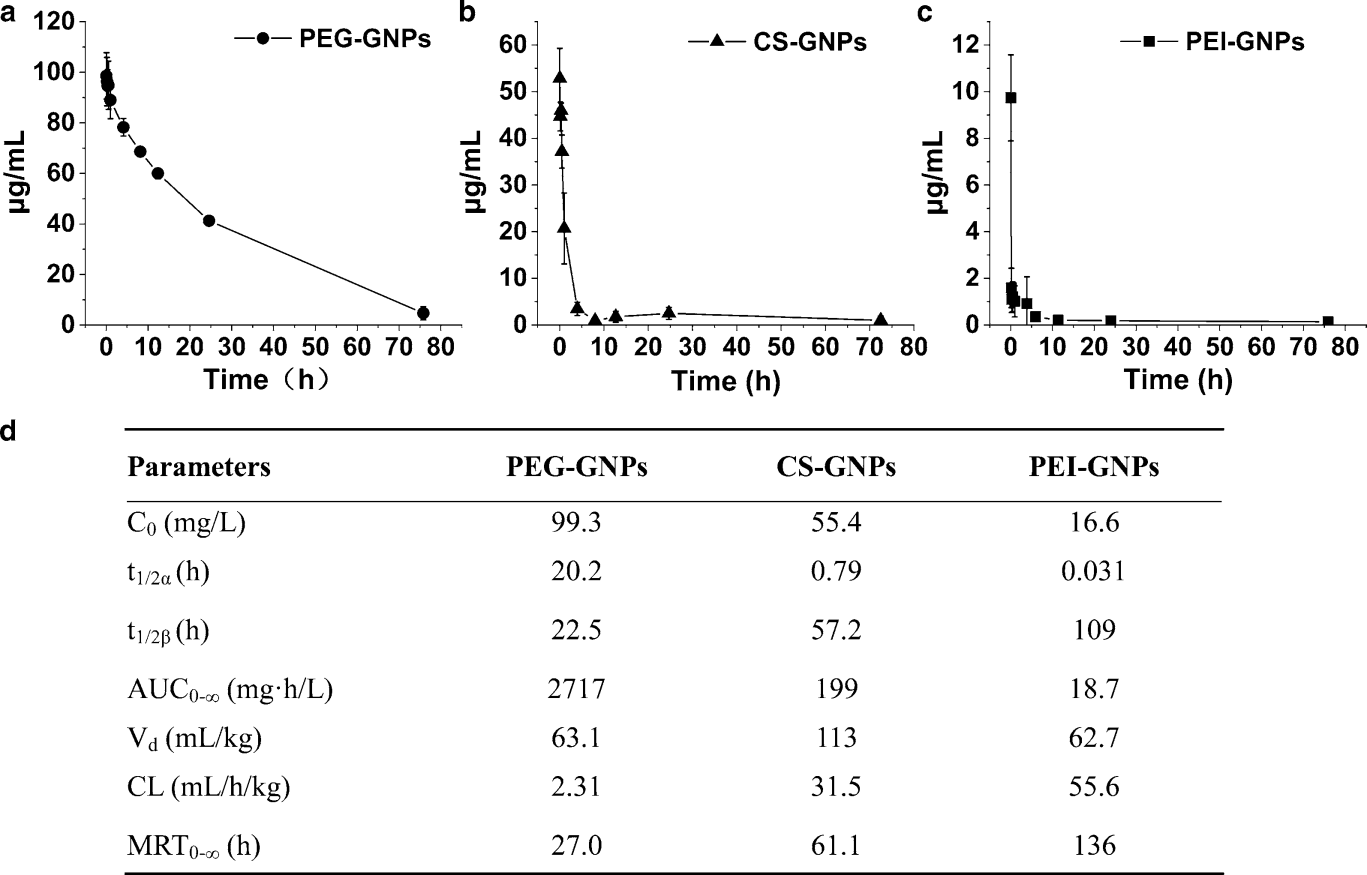
Blood kinetics of PEG-GNPs (**a**), CS-GNPs (**b**), and PEI-GNPs (**c**) after i.v. injection in mice (n = 6). *C*_*0*_ the initial concentration, *t*_*1/2α*_ plasma distribution half-life, *t*_*1/2β*_ plasma elimination half-life, *AUC*_*0-∞*_ area under the curve from zero to infinity, *V*_*d*_ apparent volume of distribution, *CL* clearance rate, *MRT*_*0-∞*_ mean residence time

The three types of functional GNPs show different pharmacokinetic profiles (Fig. 2a–c). From these profiles, their individual pharmacokinetic parameters were calculated by non-compartmental analysis (Fig. 2d). As expected, the PEG-GNPs had the longest blood circulation time, with a distribution t_1/2α_ of 20.2 h and an elimination t_1/2β_ of 22.2 h, which is similar with previously published results [25]. CS-GNPs had an intermediate t_1/2α_ and t_1/2β_, while PEI-GNPs had the shortest t_1/2α_ and the longest t_1/2β_. The concentration of PEG-GNPs remained at about 40% of C_0_ in the blood 24 h after i.v. injection, whereas, the plasma concentration of PEI-GNPs rapidly dropped from 54% C_0_ at 2 min to 6% C_0_ at 15 min after injection. Comparatively, 52% C_0_ of CS-GNPs were retained in the blood at 2 min post-injection, which then dropped to 24% at 1 h post-injection.

### Bioaccumulation of functional GNPs in organs

**Fig. 3:**
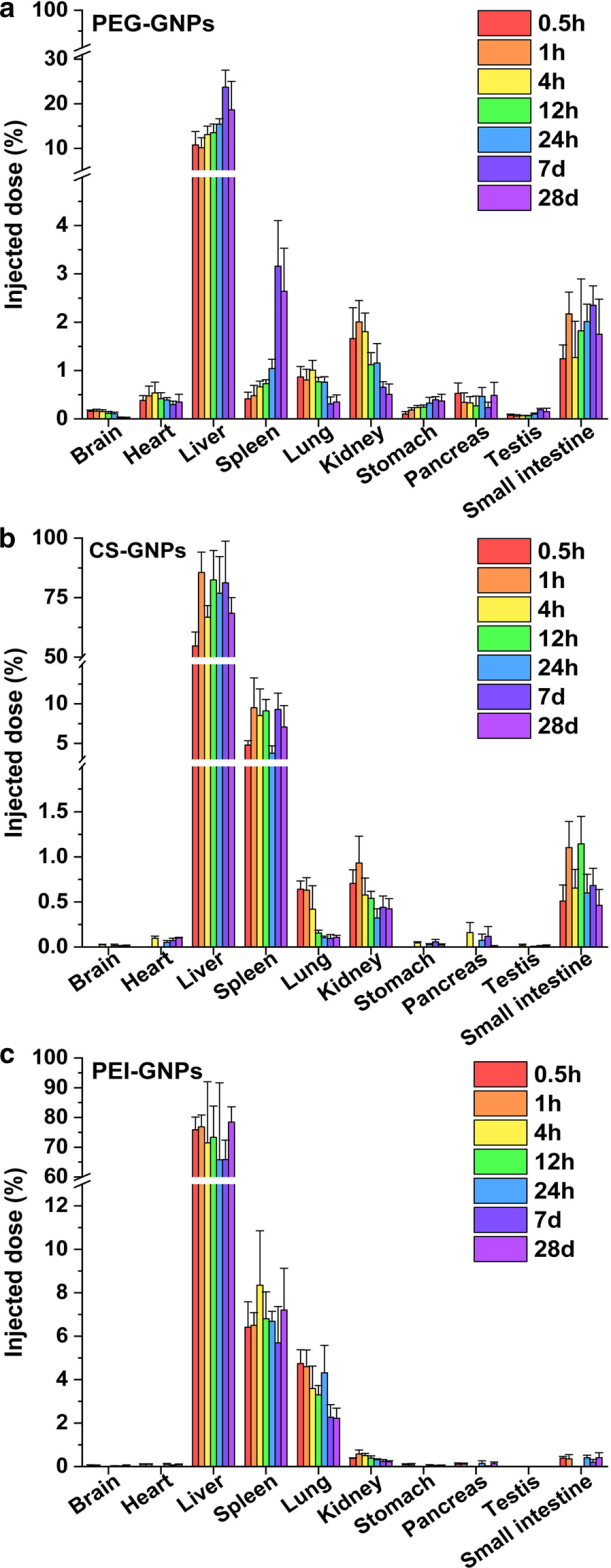
Bioaccumulation of **a** PEG-GNPs, **b** CS-GNPs, and **c** PEI-GNPs after i.v. injection

Biodistribution analysis of the three types of functional GNPs showed that they primarily accumulated in the liver, spleen, lung, kidney, and small intestine after i.v. injection (Fig. 3a–c). The obvious accumulation of PEG-GNPs in the liver was observed as early as 0.5 h post-injection and reached its highest level at day 7, from which there was no significant decrease at 28 days post-injection. Only 2% of the injected PEG-GNPs were found in the kidneys at 1 h post-injection. The CS-GNP and PEI-GNP treated mice showed a large amount of GNP accumulation in the liver and spleen. Up to 84% of injected CS-GNPs accumulated in the liver at 1 h post-injection. At day 28, nearly 68% of the CS-GNPs remained in the liver, and about 5–10% of the CS-GNPs had accumulated in the spleen. 76% of the PEI-GNPs accumulated in the liver as early as 0.5 h post-injection, and remained at that level 28 days after injection. The spleen showed consistent accumulation of 6–8% of the injected PEI-GNPs until 28 days post-injection. In the lungs, there was relatively higher accumulation of PEI-GNPs compared with PEG- and CS-GNPs treated mice. Notably, only relatively small amounts of PEI-GNPs were found in the kidney; the highest amount was 0.5% of the injected dose at 1 h post-injection. Furthermore, we compared the bio-distribution of Cit-GNPs with PEG, CS, and PEI coated GNPs (Additional file 1: Figure S6). The results indicate that about 89.4% injected Cit-GNPs was distributed in the liver at day 7 post-injection, obviously higher than that in the CS-GNP PEI-GNP, and PEG-GNP treated mice. To explore the effects of surface chemistry on the sub-organ transfer, clearance and toxicity of functional GNPs, the PEG, CS, and PEI coated GNPs were mainly investigated in the later studies.

### Excretion of functional GNPs

PEG-GNPs, CS-GNPs, and PEI-GNPs had significantly different excretion profiles in urine and feces after i.v. injection in mice (Additional file 1: Figure S7). PEG-GNPs were excreted more in feces and urine after i.v. injection than CS-GNPs and PEI-GNPs. The cumulative elimination of PEG-GNPs via feces and urine increased rapidly at initial 120 h and then increased slowly after 168 h post-injection. PEG-GNPs were excreted about 15.3% via feces and 2.7% via urine within 14 days (336 h). At day 28 post-injection, almost no Au signal was detected in urine and feces. However, for CS-GNP and PEI-GNP treated mice, within 14 days, the total excretion of GNPs via feces was 4.0% and 2.6%, respectively, which was significantly lower than that of PEG-GNP mice. Urinary excretion of CS-GNPs and PEI-GNPs was very low, which was less than 0.08% and 0.4%, respectively.

### Sub-organ transfer of functional GNPs

**Fig. 4:**
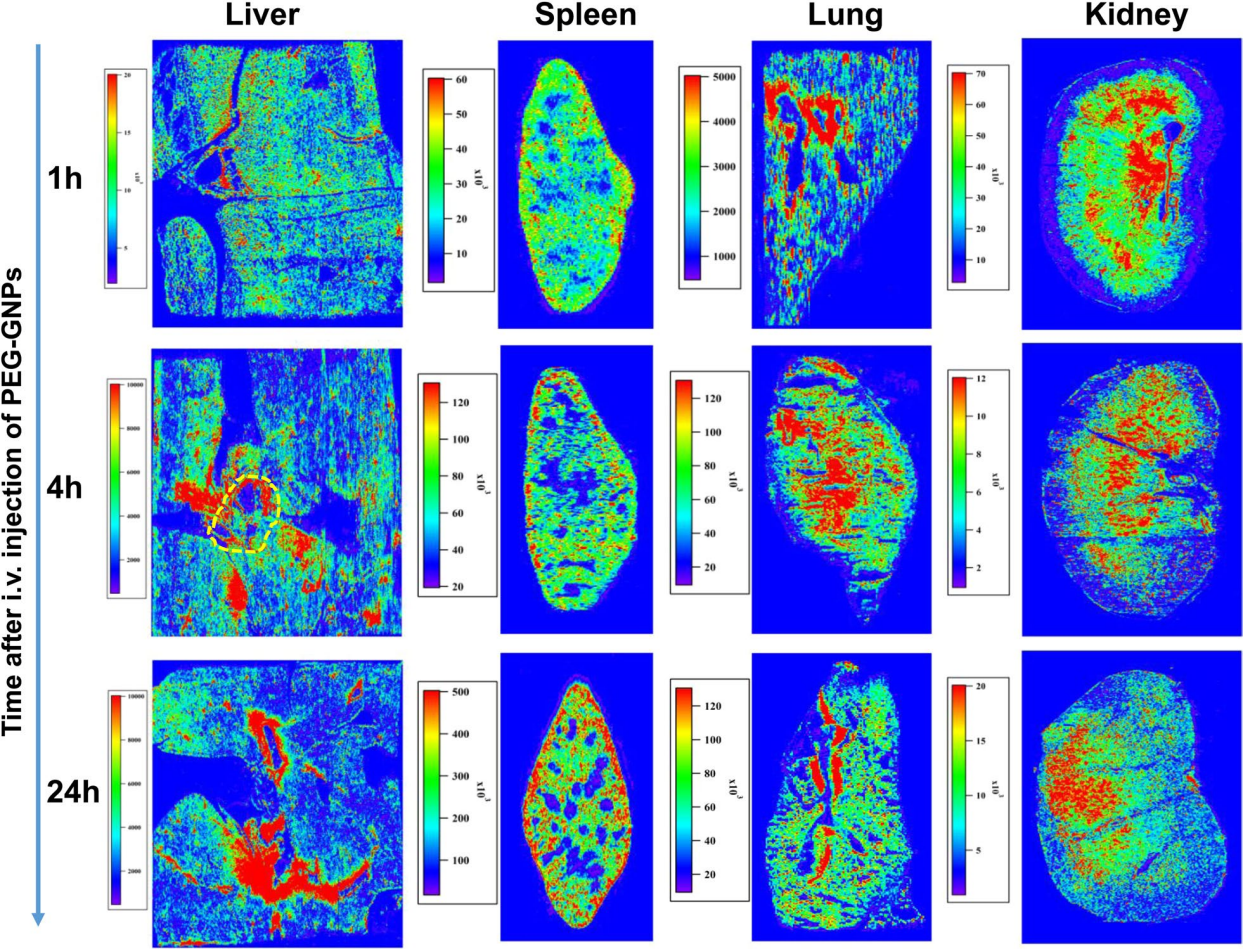
LA-ICP-MS gold images showing transportation and intra-organ distribution of PEG-GNPs in the liver, spleen, lung, and kidney. The portal triad areas were marked by the yellow dashed circle

To further investigate how the GNPs were transferred and cleared from the organs, LA-ICP-MS imaging analysis was performed to track GNPs in the liver, spleen, lung, and kidney of Wistar rats at 1, 4, and 24 h after i.v. injection (Fig. 4).

We imaged the portal area of the liver to determine whether PEG-GNPs in the blood stream could pass through the liver sinusoids and enter into the liver parenchyma. We found that high Au signal intensity accumulated in the region surrounding the portal area, and then increased markedly over time after injection. In addition, more Au accumulated near the portal region than near the central vein.

LA-ICP-MS imaging in the spleen showed that PEG-GNPs quickly entered the splenic parenchyma at 1 h after injection and increased over time. PEG-GNPs perfused from the red pulp near the white pulp region and gradually deposited in the red pulp, which is located near the marginal region of the spleen. At 24 h post-injection, a significant amount of PEG-GNPs had entered the marginal zone and the red pulp and had primarily deposited in the marginal region of the spleen. The Au images of the PEG-GNP injected mouse lung showed that the PEG-GNPs accumulated in the regions near blood vessels.

LA-ICP-MS imaging of PEG-GNPs showed very clear transfer and clearance pathways in kidney. The Au image at 1 h post-injection revealed that PEG-GNPs were quickly transported via the blood steam from the renal artery to the renal pyramids and primarily accumulated in the renal pelvis. At 4 h after i.v. injection, the intensity of the Au signal in the renal pelvis decreased, but high Au levels were broadly seen in the renal pyramids region. At 24 h post-injection, high Au signal was concentrated in the renal medulla and the renal cortex.

### Transfer and clearance of functional GNPs in organ microarchitecture

**Fig. 5:**
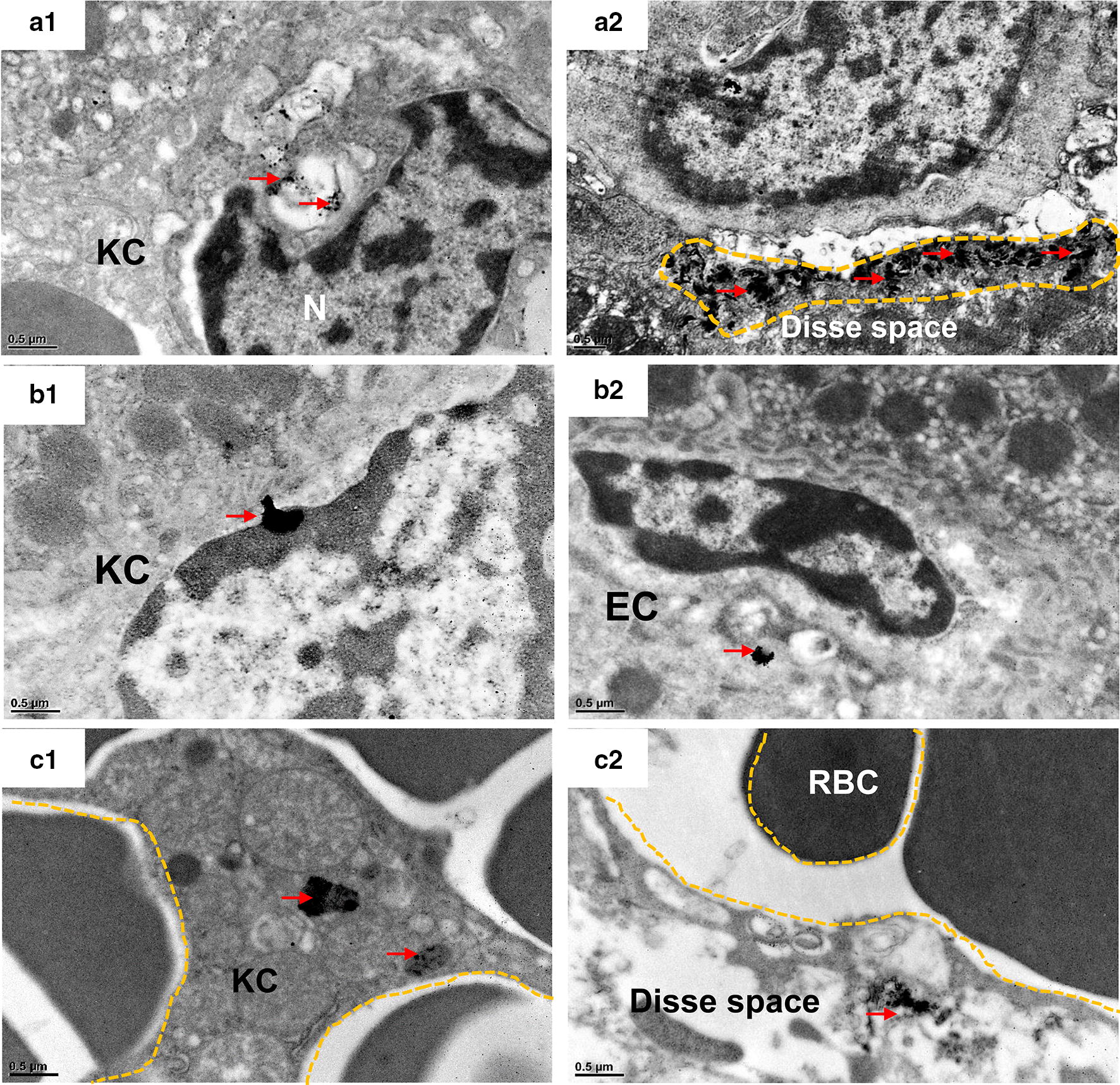
TEM images of mouse liver at 1 h after i.v. injection of functionalized GNPs. PEG-GNPs were deposited in the cytosol of KCs (**a1**) and Disse space (**a2**); CS-GNPs were deposited in the cytosol of KCs (**b1**) and cytosol of ECs (**b2**); PEI-GNPs were deposited in the cytosol of KCs (**c1**), space of Disse (**c2**). KC: Kupffer cells; EC: endothelial cells. Clusters of GNPs were indicated by red arrows

**Fig. 6:**
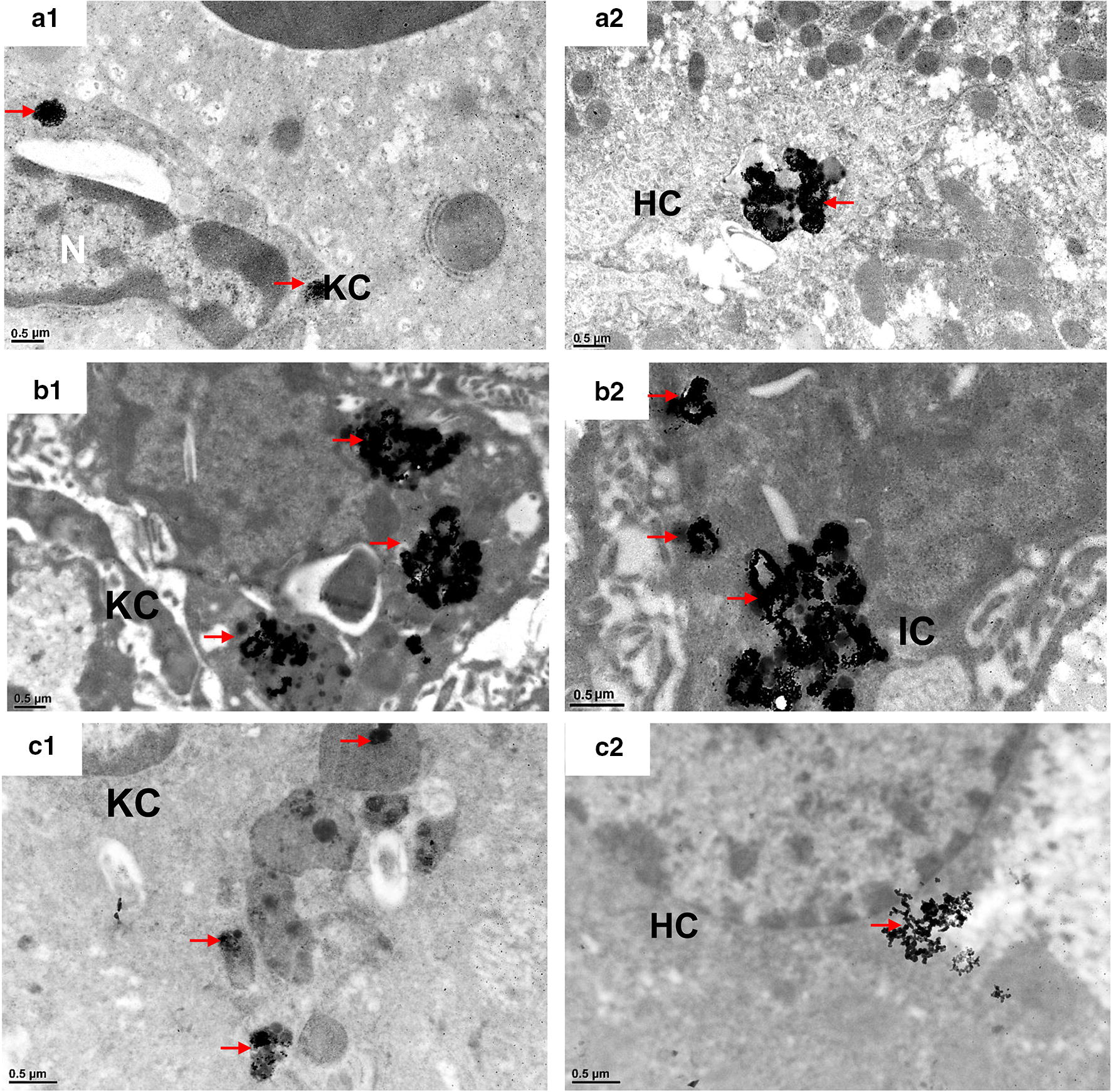
TEM images of mouse liver tissues at 28 days after i.v. injection of functionalized GNPs. PEG-GNPs were deposited in KCs (**a1**) and the secondary lysosomes of hepatocytes (**a2**); CS-GNPs were deposited in KCs (**b1**), in Ito cells in Disse space (**b2**); PEI-GNPs were deposited in secondary lysosomes of KCs (**c1**) and cytosol of HC (**c2**). *KC* Kupffer cells, *EC* endothelial cells, *IC* Ito cells, *HC* hepatocyte. GNP deposition was indicated by red arrows

**Fig. 7:**
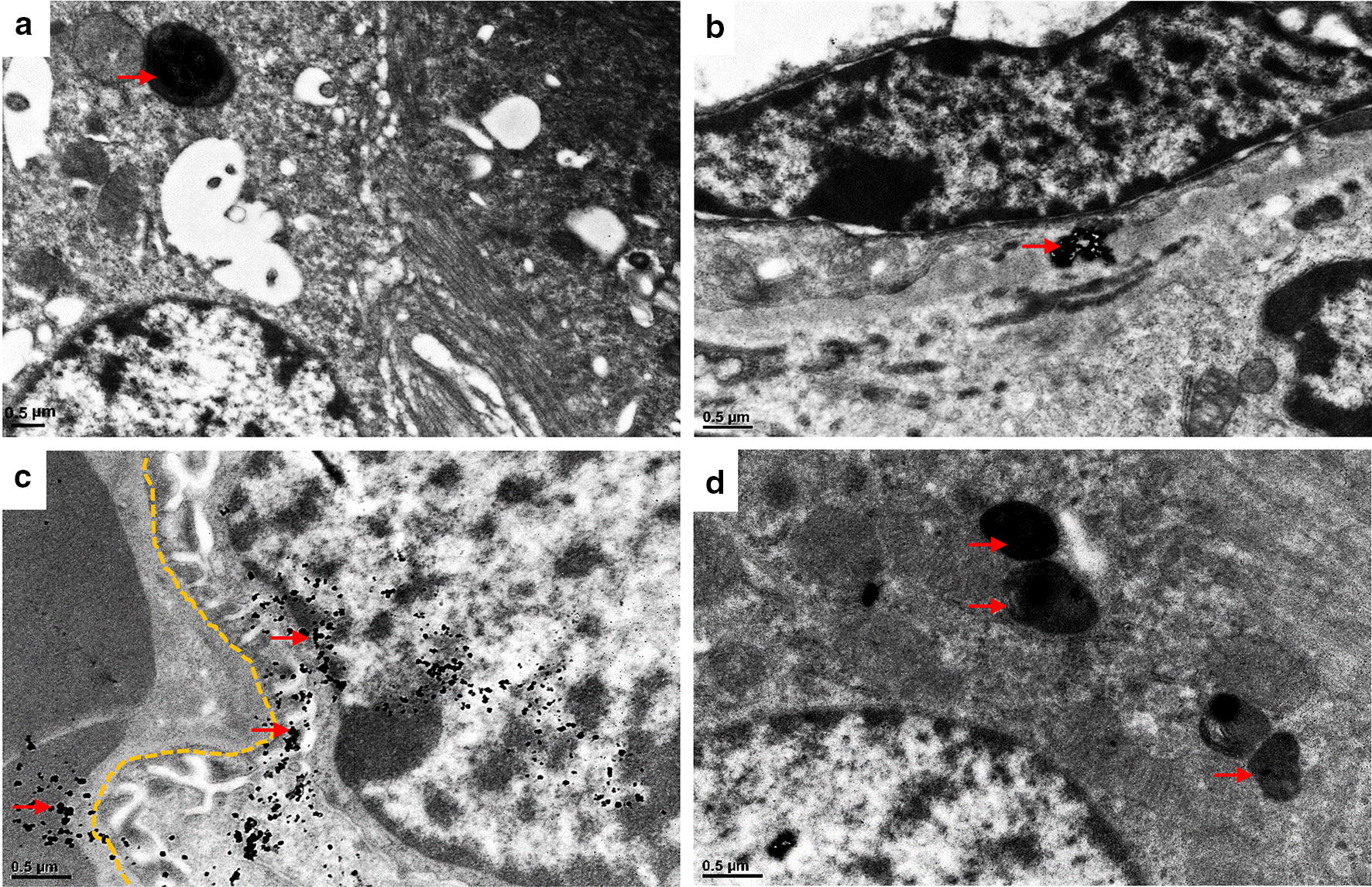
TEM images of mouse kidney at 1 h after i.v. injection of functionalized GNPs. A small amount of PEG-GNPs (red arrows) were deposited in the lysosomes of renal tubule epithelial cells (**a**) and tubulointerstitium (**b**). A lot of PEI-GNPs (red arrows) were deposited in podocytes (**c**) and the lysosomes of renal tubule epithelial cells (**d**). *GBM* glomerular basement membrane

We used TEM to investigate GNP transfer within the liver (Figs. 5, 6, Additional file 1: Figure S8–S10), spleen (Additional file 1: Figure S11), and kidney (Fig. 7). TEM imaging in mouse liver showed that a small amount of PEG-GNPs were engulfed by KCs and deposited in the cytosol at 1 h after PEG-GNP injection (Fig. 5a1). Moreover, a large amount of clustered PEG-GNPs were deposited in the Disse space (Fig. 5a2 and Additional file 1: Figure S8), indicating that the particles had passed the fenestrated (100–200 nm) hepatic sinusoidal endothelium. However, after CS-GNP injection, a large amount was taken up by KCs and ECs and induced vascular degeneration of ECs (Fig. 5b1, b2 and Additional file 1: Figure S9B1 & B2), while a little amount of agglomerated/aggregated CS-GNPs was found in hepatocytes at 1 h post-injection (Additional file 1: Figure S9 C1, C2). After PEI-GNP administration, a large amount of particles were observed in KCs (Fig. 5c1) and a little was found in Disse space and hepatocytes (Fig. 5c2, Additional file 1: Figure S10), demonstrating that a little PEI-GNPs are capable of crossing the endothelial barrier after i.v. injection and inducing hepatocyte swelling. However, PEI-GNPs induced hepatic sinus dilation and red blood cells across Disse space were observed (Additional file 1: Figure S10A). At day 28 post-injection, some PEG-GNPs were still deposited in the lysosomes of KCs (Fig. 6a1), while more clustered PEG-GNPs had accumulated in hepatocytes, suggesting PEG-GNPs were transported from Disse space into hepatocytes (Fig. 6a2). Additionally, large amounts of clustered CS-GNPs were found in KCs (Fig. 6b1) and hepatic stellate cells (HSC) (Fig. 6b2) in Disse space and induced HSC proliferation (Fig. 6b2). Little amount of PEI-GNPs were found in ECs and HCs (Fig. 6c1, c2). In the spleen, considerable amounts of PEG-GNPs were found to pass through the splenic endothelial cells and deposit in the splenic sinus (Additional file 1: Figure S11). This is described in more detail in additional material.

In the kidney, a small amount of PEG-GNPs were found deposited in the tubulointerstitium and were taken up into the lysosomes of renal tubule epithelial cells (Fig. 7a, b), demonstrating renal clearance of PEG-GNPs. We observed that PEI-GNPs accumulated in podocytes, with some across the glomerular basement membrane (GBM) trapped in the lysosomes of renal tubule epithelial cells (Fig. 7c, d). Few CS-GNPs were observed in kidney.

### Organ histology after i.v. injection of functional GNPs

Histological examinations of the liver, spleen, lung, and kidney were performed at day 28 post-injection. Severe swelling of hepatocytes was found in PEG-, CS-, and PEI-GNPs administrated mice. No obvious histological changes were found in the spleen and lung. In the kidney, we observed a mild expansion of microvessels in the renal interstitium (RI) and proteinaceous cast formation in the renal tubule (RT) in all GNP-treated mice (Additional file 1: Figure S12).

The serum biochemical profiles of the functional GNP treated mice were, to some degree, consistent with the histological observations in the liver and kidney (Additional file 1: Figures S13, S14). In PEG-GNP treated mice, at day 7 post-injection, the levels of serum total protein (TP), serum albumin (ALB) and the albumin to globulin ratio (A/G) were significantly elevated (*p* < 0.01); at day 28, only toal bilirubin (TBIL) significantly increased (*p* < 0.01), indicating the stress responds to the treatment. For CS-GNP mice, the levels of aspartate aminotransferase (AST, *p* < 0.05) and TBIL (*p* < 0.01) significantly increased at day 7 post-injection, however, at day 28, the levels of AST (*p* < 0.05) still kept significant elevation. In PEI-GNPs treated mice, the level of AST (*p* < 0.01) significantly increased and TBIL (*p* < 0.05) significantly decreased at day 7 post-injection, at day 28, the levels of TP (*p* < 0.01) and TBIL (*p* < 0.05) was found statistically elevated. These results indicated that the CS-GNPs and PEI-GNPs treatment could slightly impair liver function.

The serum biochemical alteration of creatinine (CREA), blood urine nitrogen (BUN) and uric acid (UA) levels may provide some evidence for the evaluation of kidney function. At day 7 post-injection, serum CREA level (*p* < 0.01) in PEI-GNP and UA (*p* < 0.01) in CS-GNP mice was found significant increase. At the day 28 post-injection of PEG- and PEI-GNPs, the levels of CREA (*p* < 0.01) and BUN (*p* < 0.05) in mouse serum significantly decreased, and UA in PEG-GNP mice significantly increased (*p* < 0.05).

### Liver gene expression changes after functional GNP i.v. injection

**Fig. 8:**
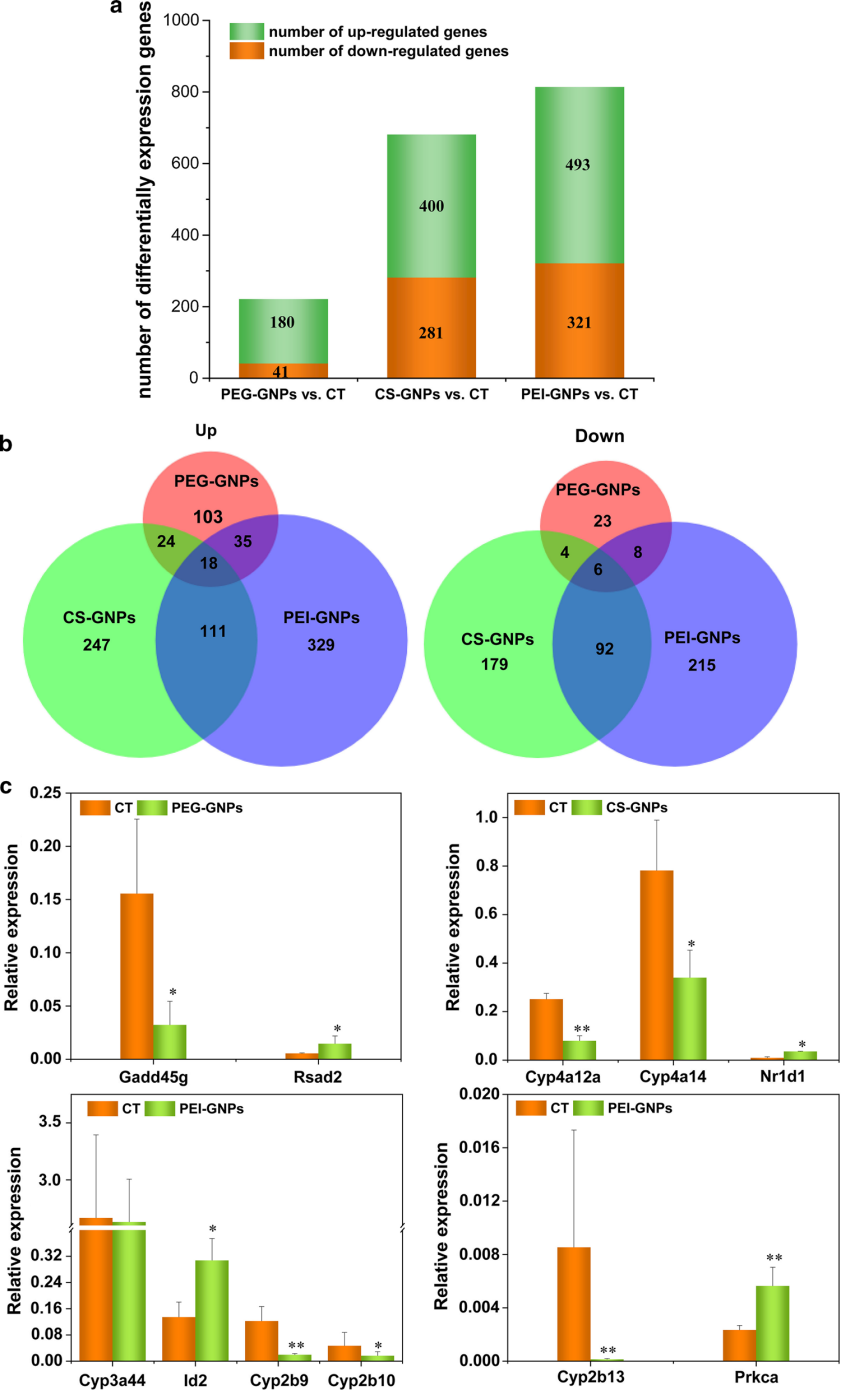
Differentially expressed genes (DEGs) profile in the liver of PEG-GNP, PEI-GNP, and CS-GNP injected mice compared with vehicle control mice. n = 3. **a** The number of DEGs in the liver of treated mice compared with vehicle control mice. **b** Venn diagram of DEGs between the treated and vehicle control mice. **c** RT-QPCR validation of selected genes in the livers of mice exposed to functionalized GNPs. **p < 0.01, *p < 0.05 (all vs. CT)

We investigated changes in liver gene expression in response to functional GNP administration at 7 days post injection using Agilent microarrays. Liver gene expression was significantly altered after single injections of PEG-GNPs, CS-GNPs, and PEI-GNPs (Fig. 8). In total, 221 (180 up-regulated and 41 down-regulated), 681 (400 up-regulated and 281 down-regulated), and 814 (493 up-regulated and 321 down-regulated) unique differentially expressed genes (DEGs) were identified after PEG-GNP, CS-GNP, and PEI-GNPs injection, respectively (Fig. 8a). These DEGs were represented by 56,745 probes at p < 0.05 and had a fold change of at least ± 1.5. Among the up-regulated genes, only 18 genes overlapped among PEG-GNP, CS-GNP, and PEI-GNP groups, but 111 genes overlapped between the CS-GNP and PEI-GNP groups (Fig. 8b). The ten most up-regulated and down-regulated genes are listed in Additional file 1: Tables S2–S4. In PEG-GNP treated mice, most of the DEGs were involved in different biological processes such as protein kinase activity, MAPK activity, and T cell differentiation and activation. The top DEGs in CS-GNP mice were related to molecular functions such as alkane 1-monooxygenase activity, icosatetraenoic acid, and long-chain fatty acid, and with biological processes such as B cell differentiation and platelet aggregation. In the PEI-GNP group, the top DEGs were generally involved in biological processes including epoxygenase P450 pathway, photoperiodism, regulation of histone deacetylation, and nonmotile primary cilium assembly, and with molecular functions such as arachidonic acid epoxygenase activity, arachidonic acid monooxygenase activity, mitogen-activated protein kinase binding, and oxidoreductase activity.

The DEGs that were highly expressed and involved in important cell processes or metabolic pathways were selected for further validation by RT-qPCR. *Gadd45g*, a stress sensor with multiple biological implications that may promote hepatocyte survival in response to environmental stresses [27], was confirmed as down regulated in the PEG-GNP treated group (Fig. 8c). The up-regulation of *Rsad2* suggests that PEG-GNP treatment could induce innate immune responses [28]. Down regulation of *Cyp4a12a* and *Cyp4a14* was observed in CS-GNP treated samples, indicating that steroid and lipid metabolism was affected [29, 30]. In PEI-GNP treated samples, the levels of *Cyp2b genes (Cyp2b13*, *Cyp2b9*, and *Cyp2b10) were significantly decreased, indicating that* PEI-GNP *i.v. injection impacted synthesis or metabolism of cholesterol, steroids, and other lipids.*

## Discussion

Here we explored how surface chemistry influences the intra-organ distribution, transfer, and excretion patterns of three types of functional GNPs (PEG-GNPs, CS-GNPs and PEI-GNPs) in vivo. The surface chemistry varied depending on the attached ligand and the final structural mode. Our study showed that the differences in surface chemistry induced dramatically different effects on the biodistribution, intra-organ transfer, and elimination profiles of these GNPs in mice. We propose that the surface chemistry induced dispersion stability of GNPs is one of the main mechanisms underlying functional GNP interactions in vivo.

The GNP surface chemistry-mediated biodistribution profiles are likely related to blood flow dynamics, cellular phenotype in the MPS, and how the GNPs agglomerate in the biological environment. The long circulatory time of PEGylated GNPs, an expected feature, demonstrated the ability of PEG to minimize opsonization and clearance by MPS sequestration. Within organs, the liver and spleen have been reported to possess a high arterial and venous blood flow but slow blood flow (200–800 µm s^−1^) in liver or splenic sinusoids. A prolonged retention time within the sinusoids would provide more opportunity for intracellular uptake [31]. The non-parenchymal liver cells, namely KCs and liver sinusoidal ECs, were major sites of NP capture, likely because they are first to interact with GNPs when they enter the liver and are highly phagocytic. However, a considerable amount of the well-dispersed PEG-GNPs were not captured by KCs or ECs, but remained in the circulation or were extravasated into Disse space via hepatic sinusoidal fenestrated endothelium (fenestrations approximately 100–200 nm in diameter) and eventually taken up by hepatocytes. Growing evidence demonstrates that some nanomaterials (TiO_2_, SiO_2_, Au, and Ag) can induce endothelial leakiness (EL), subsequently extravasate from the capillaries into interstitial space via widening endothelial gaps that could be tens to hundreds of micrometers, large enough for even whole cells to traverse [32–34]. Our study suggests that GNP extravasation through the endothelial barrier is dependent on their surface chemistry. PEG-GNPs are extravasated into Disse space through the endothelial barrier without obvious damage to ECs. On the other hand, the large agglomerated CS-GNPs and PEI-GNPs in KCs and ECs largely limited their exocytosis and recirculation or hepatocyte entry, resulting in long-term accumulation in the liver [35]. Furthermore, a small amount of CS-GNPs and PEI-GNPs were found to cross the endothelial barrier, which induced ECs damage and resulted in an endothelial gap too large for even red cells to traverse. Recent work has revealed that nanomaterial-induced endothelial leakiness (NanoEL) could favour intravasation of surviving cancer cells into the surrounding vasculature and subsequent extravasation into other tissues, accelerating metastasis [36]. Thus, NanoEL is a double-edged sword that may promote drug permeability into the tumor site, while potentially accelerating the extravasation of cancer cells. To harness the benefits of EPR effects in developing cancer nanomedicines and other nanobiotechnology, tuning the surface chemistry of NMs in order to control the size of the endothelial gap and selectively pass NM loaded drugs could be a feasible and effective strategy.

Additionally, the different GNP sequestration patterns in the liver impacts their elimination. Previous studies have shown that i.v. administered NP clearance from the circulation and elimination from the body generally occur via two main routes, renal and hepatobiliary excretion [37]. The liver plays a central role in the removal of endogenous compounds from the blood. We observed that the hepatobiliary route was the major elimination pathway for GNPs in this study. The elimination of exogenous NPs via the hepatobiliary route generally included NPs entering the liver sinusoid, passive diffusion into hepatocytes from Disse space, transportation into the bile, and subsequent removal via the intestine (where potential reabsorption is still unknown) in feces [37]. Once a compound is inside a hepatocyte, it can be transported into bile either unchanged or as more hydrophilic metabolite after phase I and/or phase II biotransformations. This may explain how the hydrophilic and well-dispersed PEG-GNPs facilitated excretion in feces. However, the interactions of agglomerated CS-GNPs and PEI-GNPs with liver non-parenchymal cells (*e.g.*, KCs and ECs) impeded the elimination of GNPs via the hepatobiliary route. Particularly, due to the formation of larger agglomeration/aggregation of PEI-GNPs and CS-GNPs in the physiological environment, less PEI-GNPs and CS-GNPs were excreted in feces, further demonstrating that more particle agglomeration/aggregation could induce less elimination from the liver.

The renal pathway for NP elimination is strictly limited to particles size smaller than 6 nm due to the pore size limitation of glomerular filtration in the kidney [38]. The filtration of intermediate-sized nanomaterials, i.e. between 6 and 8 nm, is dependent on the nanomaterial surface, surface charge, and the vascular physiological state [39]. More PEG-GNPs were eliminated in the urine than CS-GNPs and PEI-GNPs because of their well-dispersed individual particle state. In addition, a slight vasodilation of the glomerular capillary has been observed in mouse kidney during excretion, demonstrating a temporal change in the state of the capillary that could increase vascular permeability and facilitate GNP extravasation. Very little CS-GNPs or PEI-GNPs were excreted in the urine. This could be attributed to the the opsonization or protein-corona formation that increased size of CS-GNPs and PEI-GNPs in physiological microenvironment, which is beyond the size threshold of glomerular filtration [38].

The mechanisms underlying the difference in intra-organ distribution and excretion of the three types of functional GNPs need further investigation. When functional GNPs are exposed to the biological environment, their dispersion stability and “protein corona” formation have a great impact on their biological fate in vivo. The PEGylaed GNPs showed good dispersion stability and resistance to protein adsorption in vivo. Moreover, the well-dispersed and negatively charged PEGyled GNPs are quite stable in the vascular flow, and favour the interactions with ECs that facilitates PEG-GNP crossing of the hepatic and splenic sinusoids and organ elimination. In contrast, the cationically charged CS-GNPs and PEI-GNPs tended to agglomerate and adsorb serum proteins in vivo. Previous work showed that cationic GNPs had the propensity to agglomerate and be retained in the cells for a relatively long time after phagocytosis, resulting in long-term tissue accumulation [35]. “Protein corona” formation on NP surfaces considerably affects their blood circulation time, biodistribution profile, transfer, and interactions with cells [40, 41]. The surface chemistry of NPs also plays an important role in regulating protein adsorption and subsequent cellular adhesion [42]. In the present study, more blood proteins (such as BSA, IgG, and Tf) conjugated with cationically charged CS-GNPs and PEI-GNPs as compared to negatively charged hydrophilic PEG-GNPs. This caused the size of the GNPs (particularly PEI-GNPs) to increase close to the optimal size (300 nm–1 µm) for macrophage uptake [43, 44], thus influencing their bio-distribution. The well-dispersed PEG-GNPs adsorbed less proteins, were less internalized by KCs and ECs, could transfer within organs, and thus could be rapidly eliminated via hepatobiliary excretion [45]. Therefore, the surface chemistry mediated dispersion stability of GNPs influences their intracellular uptake and retention time, intra-organ transfer, and elimination.

The long retention time of GNPs in the liver increases the likelihood of nanoparticle-mediated chronic toxicity via inflammation or immunological responses. Our microarray analysis showed that i.v. injection of CS-GNPs or PEI-GNPs in mice induced more significant changes in liver gene expression than PEG-GNPs. For instance, PEG-GNP i.v. administration led to significant changes in stress-response related genes (up-regulated: *Map2k3*, *Thbs1*, and *Map2k7*; down-regulated: *Map2k6* and *Gadd45d*) and inflammation-associated genes (up-regulated: *S100a8*, *S100a9*, and *Ccr3*). However, the CS-GNPs and PEI-GNPs caused down-regulation of many cytochrome P450 related genes (especially PEI-GNP treatment) and up-regulation of inflammation-associated genes (*S100a8*, *S100a9*, and *Alox5ap* in CS-GNP treated mice, *Htra4* and *Cyr61* in PEI-GNP treated mice). Previous studies have indicated that during inflammatory processes, some circulating proinflammatory cytokines, such as IL-1β, TNF-α, and IL-6, trigger the down-regulation of hepatic P450 genes [46]. The gene expression microarray and RT-qPCR analyses provide a snapshot of the transcriptional activity in the liver after a considerable amount of agglomerated CS-GNPs or PEI-GNPs were phagocytized by KCs. This may have induced the release of cytokines, chemokines, reactive oxygen species, and an array of inflammatory mediators, leading to down-regulation of cytochrome P450 gene expression in hepatocytes via KC mediated pathways, similar to previously described mechanisms [47]. In addition, the liver sinusoidal endothelial cells (LSECs) generally display anti-inflammatory properties that prevent KC and HSC activation under normal physiological conditions [48]. The vascular degeneration of ECs in the CS-GNPs treated group indicated that ECs failed to effectively protect HCs, making them vulnerable to KC or EC mediated damage. These changes of gene expression are consistent with the alteration of liver histological pathology and serum biomarkers in PEG-, CS- and PEI-GNP treated mice.

## Conclusions

In summary, we have shown that surface chemistry-mediated dispersion stability plays a critical role in the intra-organ transfer, and excretion patterns of functional GNPs in vivo. The PEG-GNPs maintained dispersion properties in vivo, facilitating passage through the discontinuous liver/spleen sinusoidal endothelium or fenestrated glomerular endothelium, subsequently penetrating deep into the organ and finally being eliminated via the hepatobiliary or renal route. The stability of the GNP surface ligands in vivo influenced protein adsorption, agglomeration/aggregation, and interaction with different cell types within organs. The Au–S bond between the PEG ligand and the GNP is highly stable and likely reduces protein adsorption in a physiological environment, keeping PEG-GNPs well-dispersed in vivo. However, some PEI ligands could detach from the GNP surfaces and/or adsorb serum proteins, leading to GNP agglomeration/aggregation in vivo and interception by KCs and ECs in MPS rich organs. This impeded their excretion via a renal or hepatobiliary route. The long-term accumulation of CS-GNPs and PEI-GNPs in the liver induced more significant hepatic damage and changes in gene expression associated with metabolic processes, immune response, and signal transduction. The study provides evidence that manipulation of NP surface chemistry could bias the NPs to certain cell types or elimination pathways in vivo, furthering the clinical translation potential of non-biodegradable nanoparticles.

## Methods

### Synthesis of functionalized GNPs

*Cit-GNPs* The Cit-GNPs was synthesized via citrate reduction of HAuCl_4_ in water. The detailed descriptions about synthesis of Cit-GNPs were given in additional material.

*PEG-GNPs* The Cit-GNPs was re-suspended in deionized water and 12.5 mg/ml PEG-5000 was added. The mixture was vortexed immediately and then incubated at 4 °C for 8 h. The PEG-GNPs was centrifuged at 16,000*g* for several times to remove the unreacted PEG polymer. The obtained PEG-GNPs was re-dispersed in saline solution and stored at 4 °C.

*CS-GNPs* The 0.75 mg/ml CS solution was added into 50 ml Cit-GNP suspension solution with vigorous stirring for 30 min at room temperature. Then the CS-GNPs were centrifuged at 16,000*g* for several times to remove the unreacted CS. The obtained CS-GNPs was re-dispersed in saline solution and stored at 4 °C.

*PEI-GNPs* The 0.40 g/ml PEI solution was added into 50 ml Cit-GNP suspension solution with vigorous stirring at room temperature for 30 min. Then the PEI-GNPs were centrifuged at 16,000*g* for several times to remove the unreacted PEI. The obtained PEI-GNPs was re-dispersed in saline solution and stored at 4 °C.

### Physical and chemical characterization of GNPs

The morphology and size of GNPs were measured by a JEM-2100F transmission electron microscopy (TEM). The hydrodynamic diameters of GNPs in medium (deionized water and saline solution) and their zeta-potential in deionized water were measured by a Malvern Mastersizer 2000 laser diffraction analyzer. The functional GNPs were also characterized by UV–Vis and Fourier transform infrared spectroscopy (FTIR) to confirm the chemical modification on the surface of GNPs. The XPS measurements were performed at 4B9B beamline of Beijing Synchrotron Radiation Facility (BSRF, Beijing, China) equipped with a Si(111) double crystal monochromator. The core level spectra, including Au 4f, C 1 s, O 1 s, and N 1 s, were recorded at the incident photon energy of 700 eV with a step size of 0.16 eV, using a hemispherical electron energy analyzer (HA150, VSW) with energy resolution better than 300 meV.

The absorption of serum protein: BSA, IgG and Tf on Cit-GNPs, PEG-GNPs, CS-GNPs and PEI-GNPs was studied by the UV–Vis, DLS and zeta potential analysis. The 500 µg/ml GNPs was incubated with 600 mg/ml BSA, 80 mg/l IgG, and 120 mg/l Tf solution for 40 min. For agarose gel electrophoresis analysis, about 12 μl (containing 5% glycerol) of the reacted solution was loaded into the wells of a 0.5% agarose gel.

### Animals and experimental design

Male CD-1 (ICR) mice (7-week old, 20 ± 1 g of body weight) and Wistar rats (6-week old, approximately 150 g of body weight) were obtained from Beijing Vital River Laboratory Animal Technology Co. Ltd. (Beijing, China). All animal experiments were reviewed and approved by the institutional animal care and use committee. The mice were housed in a conventional animal facility with 25 ± 2 °C, 60 ± 2% humidity and a regular 12 h light/dark cycle and provided standard commercial pellet diet and deionized water ad libitum. Animals were acclimatized for at least three days before experiments. Animal experiment design has been described in Additional file 1: Figure S15. All animal experiments were reviewed and approved by the institutional animal care and use committee.

For the pharmacokinetic study, 200 μl of freshly prepared PEG-GNP, CS-GNP and PEI-GNP suspension solution were intravenously (i.v.) injected via tail vein into mice at doses of 5.0, 5.0 and 0.8 μg/g body weight, respectively, of which the doses were comparable to the usually biomedical performance, including photothermal tumor therapy, bioimages and drug delivery of GNPs.

For further visualization of the sub-organ transfer and accumulation of GNPs in mice by the method of laser ablation inductively coupled plasma mass spectrometry (LA-ICP-MS), 2 ml of PEG-GNPs in saline solution were i.v. injected into the Wistar rats (n = 3) at a dose of 3.0 μg/g bw.

The mice or rats i.v. injected with saline solution were used as control group.

### Dynamic biodistribution, transfer and excretion analysis of functional GNPs in vivo

The mice were euthanized under diethyl ether-induced anesthesia at 0.5, 1, 4, 12, 24 h (n = 6), day 7 and 28 (n = 8) post-injection. The organic tissues: brain, heart, liver, spleen, lung, kidney, stomach, pancreas, testes and small intestine were weighted, collected and stored at − 80 °C for further analysis. Blood samples of mice were collected at 2, 10, 20 and 30 min; 1, 4, 8, 12, 24, and 72 h post-injection for blood-GNP clearance analysis. The serum samples at day 7 and 28 post-injection were collected and immediately sent for serum biochemistry analysis. The concentrations of Au in samples were quantitatively analyzed by ICP-MS (Thermo Elemental X7 ICP-MS). The Au content in urine and feces was normalized as a percentage of the injected dose to the digested weight of feces or volume of urine, and finally re-adjusted to the total collected weight of daily feces (1.4–2.8 g/d) or urine (1–3 ml/day).

For LA-ICP-MS image measurement, the rats were perfused with 0.9% NaCl and followed by 4% buffered paraformaldehyde in 0.1 mol/l phosphate buffered saline (PBS). After complete perfusion, the spleen, kidney, anterior caudate lobe of the liver, and superior lobe of left lung were removed immediately and stored at − 80 °C. A cryostat was used to slice the frozen tissues into 20 μm (spleen, kidney and lung) or 40 μm (liver) thick at − 20 °C. The portal region of the perfused liver was selected for the study. For spleen tissue, a cross-section sample was measured. The tissue section was put on a glass substrate. LA-ICP-MS line scans was performed to obtain the distribution of Au in sub-organs. The intensity of Au signal was normalized using ^13^C as internal standard and the images were obtained using the software Igor pro 6.10.

### Histopathological examination

Histological examinations of the liver, spleen, lung, and kidney were performed at day 28 post-injection. The organs of liver, spleen, lung and kidney of mice at day 28 post-injection were harvested and fixed in 4% formalin solution. Then the tissue samples were embedded in paraffin blocks, sectioned into 5 μm slices, and stained with hematoxylin and eosin (H&E) for histopathological examination.

### Microarray gene expression analysis

Four groups of mice (n = 3) were i.v. injected with saline, PEG-GNPs, CS-GNPs and PEI-GNPs suspension solution at the same dose as above. At the day 7 post-injection, the mice were sacrificed and the liver was collected, immediately snap-frozen in liquid nitrogen and stored in − 80 °C. Total RNA from liver tissue was extracted using RNAiso Plus reagent (Takara, Japan). The RNA integrity number (RIN) was checked by an Agilent Bioanalyzer 2100 (Agilent technologies, Santa Clara, CA, US) and the values > 7 were submitted to gene microarray test. The qualified total RNA was further purified using RNeasy mini kit (QIAGEN GmbH, Germany) and RNase-Free DNase Set (QIAGEN, GmBH, Germany).

For each GNP-treated/vehicle sample, 60 ng of total RNA was amplified and labeled with Cy3 using the Agilent Low Input Quick Amp Labeling One Color Kits, and then hybridized to Agilent SurePrint G3 Mouse Gene Expression v2 8 × 60 K microarray as manufacturer's specifications. Probe intensities were extracted using the Feature Extraction Software 12.0. Raw data were normalized by a Quantile algorithm using R/Bioconductor limma package. To detect expression differences between treated and vehicle group, a moderated T test was applied, which was generated by limma bioconductor package. Differentially expressed genes (DEGs) were selected as those with a fold change > 1.5 or < − 1.5, and *p* < 0.01 (treated vs. control mice). The enrichment analyses of DEGs were performed by Gene Ontology (GO) Consortium (GO: https://www.geneontology.org/) and KEGG public pathway resource.

### Quantitative real-time PCR assay

To confirm the gene array data, real-time quantitative PCR was performed. Total RNA was extracted from samples using TRIzol reagent (Invitrogen, USA) and then reverse transcribed with a high-capacity RNA-to-cDNA kit (Invitrogen, USA). The expression of significantly different mRNAs was determined by qPCR with SYBR Select Master Mix (Invitrogen, USA), meanwhile, GAPDH was used as an internal control. For quantitative results, the expression of each mRNA was represented as fold change by using 2^−ΔΔCt^ methods. Differences in the mRNA expression between experimental groups and control were analyzed with Student’s *t*-test. The *p* < 0.05 was considered significant. The primers used in this study are listed in Additional file 1: Table S5.

## Supplementary information

**Additional file 1.** Additional figures and tables.

## Data Availability

All data generated or analyzed during this study is available from corresponding author on reasonable request.
