# Supplementary material for: Surface chemistry governs the sub-organ transfer, clearance and toxicity of functional gold nanoparticles in the liver and kidney
Source: J Nanobiotechnology. 2020 Mar 14;18:45. doi: 10.1186/s12951-020-00599-1 (PMC7071704; doi:10.1186/s12951-020-00599-1)
Supplement: Supplementary file 1 — Additional file 1. Additional figures and tables. [file 12951_2020_599_MOESM1_ESM.docx]

**Surface Chemistry Governs the Sub-Organ Transfer, Clearance and Toxicity of Functional Gold Nanoparticles in the Liver and Kidney**

Xue Li^1,2^, Bing Wang^1*^, Shuang Zhou^1,2^, Wei Chen^1,2^, Hanqing Chen^1^, Shanshan Liang^1^, Lingna Zheng^1^, Hongyang Yu^4^, Runxuan Chu^1,4^, Meng Wang^1^, Zhifang Chai^1,5^, Weiyue Feng^1*^

^1^CAS Key Laboratory for Biomedical Effects of Nanomaterials and Nanosafety, Institute of High Energy Physics, Chinese Academy of Sciences, Beijing 100049, China

^2^University of Chinese Academy of Sciences, Beijing 100049, China

^3^ Institute of Health Sciences, Anhui University, Hefei, Anhui 230601, China

^4^ School of Environmental and Material Engineering, Yantai University, Beijing 264005, China

^5^ State Key Laboratory of Radiation Medicine and Protection, School for Radiological and interdisciplinary Sciences (RAD-X), Soochow University, Suzhou, Jiangsu 215123, China.

*Corresponding author: *E-mail address:* fengwy@ihep.ac.cn

**Materials and Methods**

**Reagents and materials**

Hydrogen tetrachloroaurate (III) trihydrate (HAuCl_4_∙3H_2_O, 99.99%) was purchased from Sinopharmacy Company (Beijing, China). Trisodium citrate dihydrate (Na_3_C_6_H_5_O_7_, 99%) was purchased from Alfa Aesar (Ward Hill, MA, USA). Methoxy PEG Thiol (mPEG-SH, M.W. 5000 ± 500, ≥ 90%) was obtained from Jenkem Technology Co. Ltd. (Beijing, China). Ethylene imine polymer [(CH_2_CH_2_NH)_n_, M.W. 10,000, ≥ 99%] was from Aladdin Chemical Co. Ltd. Glycol chitosan (G7753, degree of polymerization ≥ 400, the assay ≥ 60%, M.W. ≥ 82,084) was from Sigma Aldrich. Ultrapure deionized water was obtained from Milli-Q system (18.2 MΩ∙cm) and used in all the experiments.

**Synthesis of functionalized GNPs**

***Cit-GNPs*** The Cit-GNPs was synthesized *via* citrate reduction of HAuCl_4_ in water. Briefly, a certain amount of 1% wt. HAuCl_4_ and 0.1% AgNO_3_ solution was slightly mixed, afterwards, 2% citrate aqueous solution was added and the mixture was incubated at room temperature. Then the mixture was added into deionized water with vigorous stirring under reflux until the color of the solution changed to wine red. Finally, the Cit-GNP solution was cooled to room temperature and store at 4°C for further use.

**Results**

**Binding modes of chemical ligands to GNPs**

The PEG-GNP FTIR spectra had additional peaks at 2880 cm^-1^ (-CH_2_ stretching) and 1100 cm^-1^ (C-O-C stretching) compared with the Cit-GNP spectra. Additionally, the typical COO- peaks at 1575 cm^-1^ and 1394 cm^-1^ had disappeared, demonstrating the GNP surfaces were completely covered with PEG-SH (**Figure S2A**). The CS-GNP spectra had a representative C-O peak at 1060 cm^-1^ and a broad band at 3250-3350 cm^-1^, which is attributed to the OH and NH_2_ groups in CS (**Figure S2A**). The presence of COO- at 1570 cm^-1^ and 1392 cm^-1^ in the CS-GNPs indicated surface crosslinking via ionic interactions between positively charged chitosan and citrate anions.^1,2^ The characteristic CH_2_ bending modes were observed at 1472 cm^-1^ and 1311 cm^-1^ in PEI-GNPs (**Figure S2A**). The broadband peaks at 3300 cm^-1^ in PEI-GNPs, could be devoted to the stretching vibration of NH_2_ group and 1570 cm^-1^ to N-H bending vibration of the primary amine branch in PEI. The absence of typical COO^-^ peaks in PEI-GNPs indicates that the GNP citrate ligands were displaced by PEI. ^3^

The XPS spectra of Cit-, PEG-, CS-, and PEI-GNPs are depicted in **Figure S2-S3**. Compared with the Au 4f binding energy (BE) of 87.6 eV in Cit-GNPs, the Au 4f_7/2_ peaks in PEG-GNPs, CS-GNPs and PEI-GNPs showed a 0.1 eV increase (**Figure S2B**), indicating that the surface gold had changed from Au(0) to an Au species with a higher oxidized state (Au^δ+^). Further quantitative analysis (**Table S1**) showed that the dominant Au oxidation state of the Cit-GNPs is Au(0), with minimal charge transfer at the citrate/GNP interface. The dominant Au oxidation state of the PEG-GNPs is Au(I), suggesting that most of the citrate ligands on the GNP surface were displaced by PEG-SH. The analysis of C 1s XPS peak showed that compared with Cit-GNPs, the obvious decrease of C-C/C-H content and the appearance of C-O/C-N peaks demonstrated that PEG, CS, and PEI were successfully coated on GNPs (**Figure S3**, **Table S1**). The C-O/C-N peaks appeared in PEG-GNPs (54.3%), CS-GNPs (42.6%) and PEI-GNPs (58.5%). No COO-Au peaks were observed in the PEG-GNPs and PEI-GNPs C 1s spectra (**Table S1**), indicating that the citrate on GNPs was displaced with PEG and PEI ligands, consistent with the FTIR findings. Notably, quaternary ammonium species were observed in PEI-GNPs, indicating that oxidative coordination between PEI and GNPs had occurred.^4^

**Transfer and clearance of functional GNPs in spleen**

**Figure S11** shows the three functional GNPs in the spleen. PEG-GNPs were observed in the splenic sinus (**Figure S11A**). Particularly, little amount of well dispersed PEG-GNPs were found on the surface of red blood cells and phagocytized by macrophage (**Figure S11B**). CS-GNPs were mainly located in lysosome and cytosol of macrophage **(Figure S11C, D**). It can be seen that PEI-GNPs were adsorbed on lysosome membrane surface and cytosol in macrophages (**Figure S11E, F**).

**References**

1. Prado-Gotor R, Lopez-Perez G, Martin MJ, Cabrera-Escribano F, Franconetti A. Use of gold nanoparticles as crosslink agent to form chitosan nanocapsules: study of the direct interaction in aqueous solutions. *J Inorg Biochem* 2014;**135**:77-85.
2. Jiang CF, Zhu J, Li Z, Luo JH, Wang JS, Sun Y. Chitosan-gold nanoparticles as peroxidase mimic and their application in glucose detection in serum. *Rsc Adv* 2017;**7**:44463-69.
3. Cho TJ, Pettibone JM, Gorham JM, Nguyen TM, MacCuspie RI, Gigault J, et al. Unexpected changes in functionality and surface coverage for Au nanoparticle PEI conjugates: implications for stability and efficacy in biological systems. *Langmuir* 2015;**31**:7673-83.
4. Xue Y, Li X, Li H, Zhang W. Quantifying thiol-gold interactions towards the efficient strength control. *Nat Commun* 2014;**5**:4348.

Table S1. Electron binding energy and relative content of C with various chemical species

| **Binding energy (eV)** | COH/CH_2_ | C-C/C-H | C-S | C-O/C-N | COO-Au/O-C-O | COO(H) |
| --- | --- | --- | --- | --- | --- | --- |
| Cit-GNPs | 285.4 | / | / | / | 288.0 | 289.0 |
| PEG-GNPs | / | 286.0 | 286.6 | 287.7 | / | / |
| CS-GNPs | / | 285.6 | / | 287.0 | 288.5 | 289.9 |
| PEI-GNPs | / | 286.1 | / | 287.6 | / | 289.7 |
| **Relative content (%)** | COH/CH_2_ | C-C/C-H | C-S | C-O/C-N | COO-Au/O-C-O | COO(H) |
| Cit-GNPs |  | 91.3 | / | / | 5.2 | 3.7 |
| PEG-GNPs | / | 26.8 | 18.8 | 54.3 | / | / |
| CS-GNPs | / | 41.4 | / | 42.6 | 10.9 | 5.1 |
| PEI-GNPs | / | 38.4 | / | 58.5 | / | 3.1 |

Table S2. Top 10 GO biological process terms enriched for up-regulated and down-regulated genes in PEG-GNP treated mice compared with the controls

| **Term** | **P value** | **Enrich factor** | **Upregulated Genes** | **Down-regulated Genes** |
| --- | --- | --- | --- | --- |
| protein serine/threonine/tyrosine kinase activity | 3.52E-05 | 13.89 | Map2k3 Map2k7 | Sgk1 Map2k6 |
| ctivation of MAPK activity | 0.000159 | 7.54 | Map2k3 Thbs1 Map2k7 | Tdgf1 Map2k6 |
| alpha-beta T cell differentiation | 0.000834 | 6.79 | Batf Ikzf1 Rsad2 | Gadd45g |
| granulocyte chemotaxis | 0.001273 | 6.16 | S100a9 Thbs1 S100a8 Ccr3 |  |
| cardiac muscle contraction | 0.00139 | 6.03 | Map2k3 Atp1a3 Mybpc3 | Map2k6 |
| leukocyte chemotaxis | 0.000372 | 5.36 | S100a9 Thbs1 Cxcl10 Ccr3 S100a8 Hc |  |
| alpha-beta T cell activation | 0.003629 | 4.82 | Batf Ikzf1 Rsad2 | Gadd45g |
| striated muscle contraction | 0.004147 | 4.67 | Map2k3 Atp1a3 Mybpc3 | Map2k6 |
| positive regulation of MAP kinase activity | 0.002586 | 4.37 | Thbs1 Map2k7 Map2k3 | Tdgf1 Map2k6 |
| heparin binding | 0.006022 | 4.27 | Fgfbp3 Thbs1 Mpo Cxcl10 |  |

Table S3. Top 10 GO biological process terms enriched for up-regulated and down-regulated genes in CS-GNP treated mice compared with the controls

| **Term** | **P value** | **Enrich factor** | **Upregulated Genes** | **Down-regulated Genes** |
| --- | --- | --- | --- | --- |
| Alkane 1-Monooxygenase Activity | 0.000004007 | 30.02 |  | Cyp4a14 Cyp4a10 Cyp4a31 Cyp4a12a |
| Arachidonic Acid Binding | 0.00001076 | 22.52 | S100a9 S100a8 Alox5ap | Cyp4a14 |
| Icosatetraenoic Acid Binding | 0.00001644 | 20.01 | S100a9 S100a8 Alox5ap | Cyp4a14 |
| Negative Regulation Of Platelet Aggregation | 0.00002421 | 18.01 | Ptger3 Abat Alox12 | Serpine2 |
| Negative Regulation Of Homotypic Cell-Cell Adhesion | 0.00001543 | 13.25 | Ptger3 Abat Alox12 Cd24a | Serpine2 |
| Regulation Of B Cell Differentiation | 0.000001742 | 11.26 | Ikzf1 Prdm1 Cd24a Nfam1 Id2 | Hmgb3 Ptpn6 |
| Regulation Of Platelet Aggregation | 0.000187 | 10.6 | Ptger3 Abat Alox12 | Serpine2 |
| Long-Chain Fatty Acid Binding | 0.0002347 | 10.01 | S100a9 Alox5ap S100a8 | Cyp4a14 |
| Mrna Splice Site Selection | 0.0006263 | 7.83 | Ptbp2 | Luc7l Celf2 Luc7l3 |
| Cellular Response To Interferon-Beta | 0.0002337 | 7.51 | Irf1 Stat1 Ifit1 Ifi205 Ifi204 |  |

Table S4. Top 10 GO biological process terms enriched for up-regulated and down-regulated genes in PEI-GNP treated mice compared with the controls.

| **Term** | **P value** | **Enrich factor** | **Upregulated Genes** | **Down-regulated Genes** |
| --- | --- | --- | --- | --- |
| epoxygenase P450 pathway | 0.000005722 | 9.47 |  | Cyp2b10 Cyp2b13 Cyp2c38 Cyp2e1 Cyp2b9 Cyp2c40 Cyp2c39 |
| photoperiodism | 0.0004656 | 8.55 | Cry2 Aanat Id2 | Usp2 |
| regulation of histone deacetylation | 0.0008329 | 7.38 |  | Nipbl Jdp2 Kdm5a Lpin1 |
| mitogen-activated protein kinase binding | 0.0003439 | 7 | Atf7 Stau2 Tab1 | Plcb4 Tpr |
| oxidoreductase activity, acting on paired donors, with incorporation or reduction of molecular oxygen, reduced flavin or flavoprotein as one donor, and incorporation of one atom of oxygen | 0.0001195 | 6.96 |  | Cyp2c38 Cyp2b10 Cyp2e1 Cyp2b9 Cyp2c40 Cyp2c39 |
| arachidonic acid epoxygenase activity | 0.00001462 | 6.91 | Cyp2j9 | Cyp2c38 Cyp2b10 Cyp2b13 Cyp2e1 Cyp2b9 Cyp2c40 Cyp2c39 |
| arachidonic acid monooxygenase activity | 0.00001968 | 6.63 | Cyp2j9 | Cyp2c38 Cyp2b10 Cyp2b13 Cyp2e1 Cyp2b9 Cyp2c40 Cyp2c39 |
| insulin-like growth factor binding | 0.001385 | 6.5 | Htra4 Cyr61 | Ctgf Igfbp1 |
| aromatase activity | 0.00055 | 6.34 |  | Cyp2c38 Cyp2b10 Cyp2b9 Cyp2c40 Cyp2c39 |
| nonmotile primary cilium assembly | 0.002497 | 5.6 | Bbs2 Bbs10 Mks1 | C2cd3 |

Table S5. The primer sequences of genes

| **Gene** | **Primer Sequence** |
| --- | --- |
| Gadd45g-F | GACTGCACTGCTCTTTCAAAAAAC |
| Gadd45g-R | CACGATAGCGTCCTTTAGAAAATG |
| Rsad2-F | TCCCAGGTTTCGATCTGTTTG |
| Rsad2-R | TGTGATTGGTCGCCTGTTTATC |
| Cyp4a12a-F | ACTGGTCCTGTTCTTAGTTCTATCAAGA |
| Cyp4a12a-R | TCTGTTAGGTTGGTTGAAAATTGG |
| Nr1d1-F | ACCTTTGAGGTGCTGATGGTG |
| Nr1d1-R | GGCTCAGGAACATCACTGTCTG |
| Cyp4a14-F | GATGGTATCTCTGGGTTCTTCCA |
| Cyp4a14-R | CCTTGAACAGCACCAGAAATAGAC |
| Cyp2b10-F | GCCCAAGTCCGCACTTATCA |
| Cyp2b10-R | TCAGACCTTATACAGAGTCCATTAGCA |
| Cyp2b13-F | GGCTCTATTTTTGTGCCTCTGAGA |
| Cyp2b13-R | AAAGATGTGAGCAGCAACATACAGT |
| Cyp2b9-F | TCCACTTATCAGCTCTCCATGATC |
| Cyp2b9-R | GGCAAGTCTGTAGACATAGAAATCAGA |
| Id2-F | TTGAGTGAACCTTGTGGACTCTTTA |
| Id2-R | TAGGTTATTTCTGCCACTATACAAGAAAA |
| Prkca-F | CCTCTACTCTCCGTTTGCATGA |
| Prkca-R | GCTTGCTTCCGAACTACAATGA |
| Cyp3a44-F | TTCTCAGTGTCTGTGCAAGTTAACAT |
| Cyp3a44-R | AAACGGTGAAGGCAAATTTCC |
| Gapdh-F | TCCTGCACCACCAACTGCTTAG |
| Gapdh-R | AGTGGCAGTGATGGCATGGACT |

**
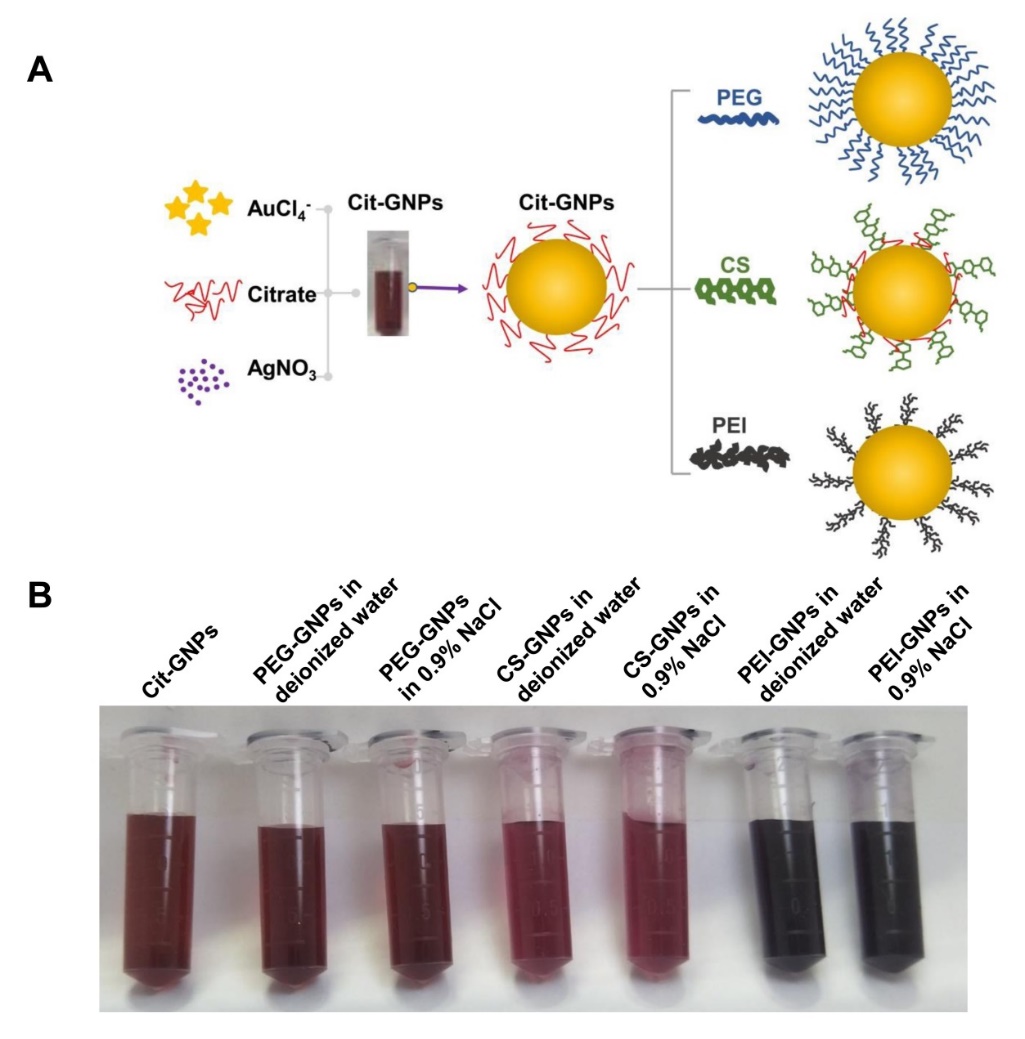
**

Figure S1. Synthesis of functionalized GNPs. (A) Schematic procedure for synthesis of Cit-GNPs, PEG-GNPs, CS-GNPs and PEI-GNPs. (B) Cit-GNPs, PEG-GNPs, CS-GNPs and PEI-GNPs dispersed in deionized water and 0.9% NaCl solution.


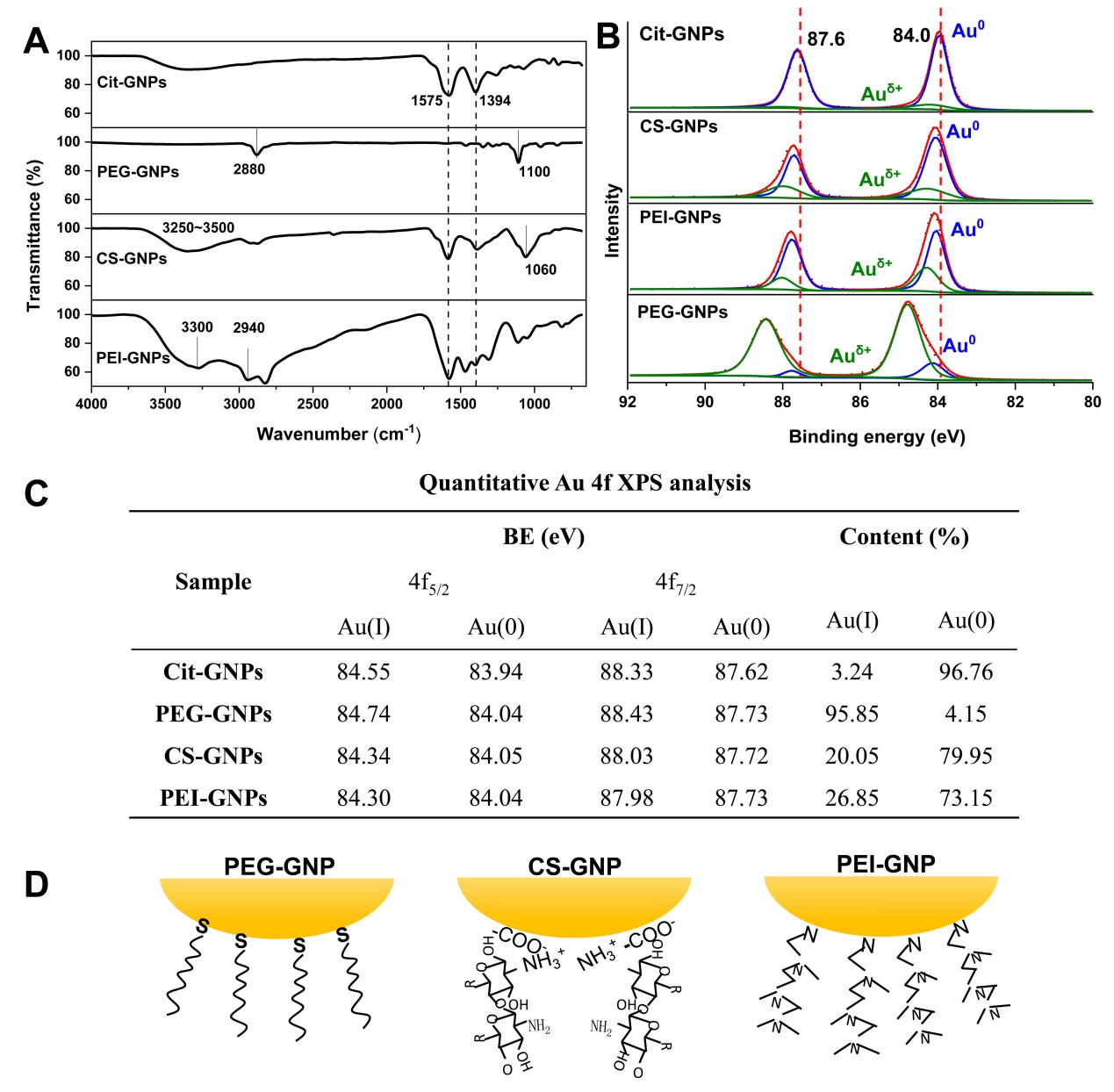


Figure S2. FTIR and XPS analysis of chemical ligands on functional GNP. (A) FTIR spectra of functionalized GNPs. Dashed lines indicate typical peaks of COO- band for citrate ligand. The FTIR spectra indicates the citrate ligands in Cit-GNPs were displaced by PEG or PEI in PEG-GNPs and PEI-GNPs. The COO- presenting in CS-GNPs reveals the surface crosslinking interaction between chitosan and citrate anions. (B) Au 4f core-level XPS spectra of functionalized GNPs. The increase of 0.1 eV of Au 4f_7/2_ peak in PEG-GNPs, CS-GNPs and PEI-GNPs indicate that the surface gold changed from Au(0) to a higher oxidized state (Au^δ+^). (C) Quantitative Au 4f XPS analysis.

**
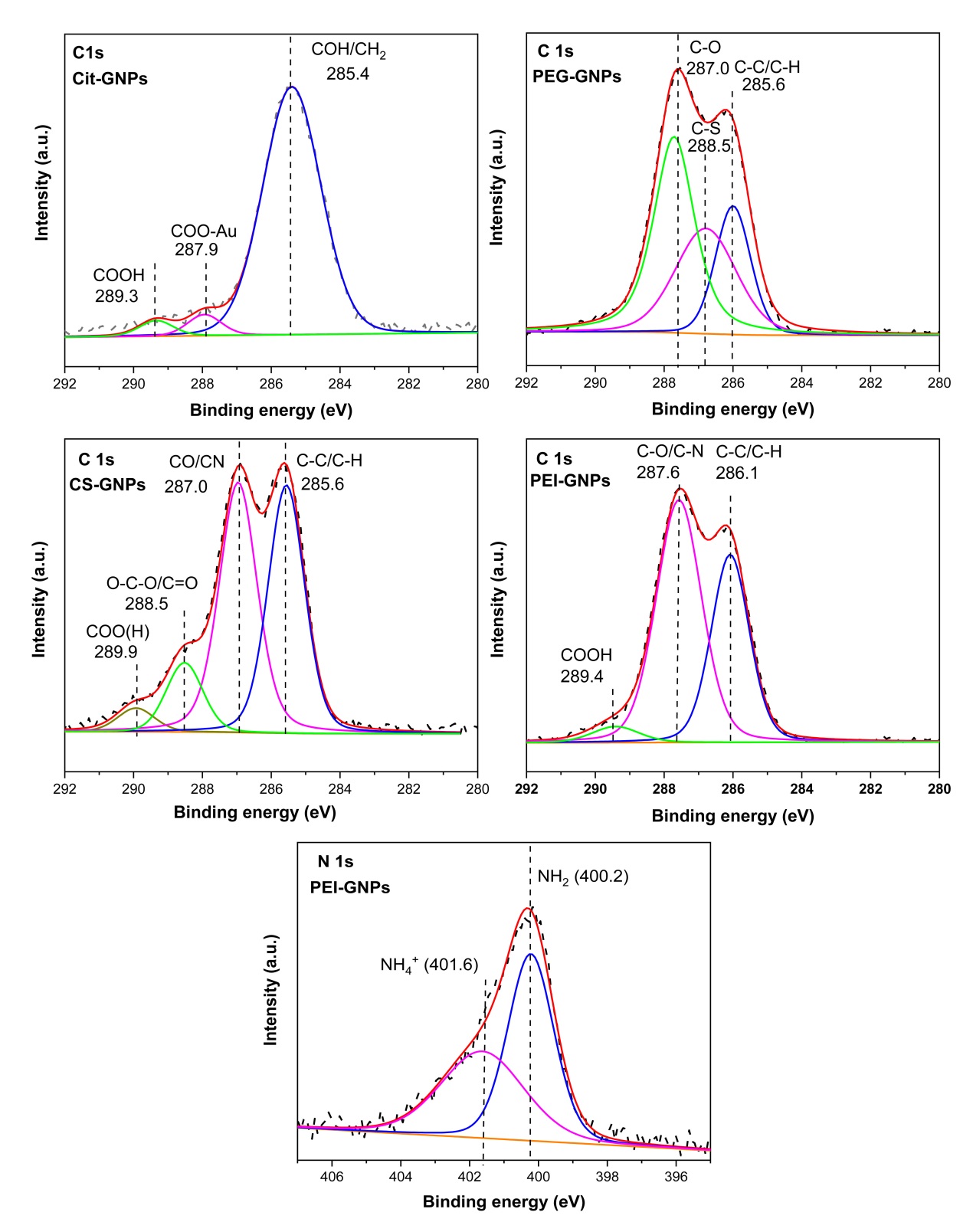
**

Figure S3. C 1s and N 1s XPS spectra.

**
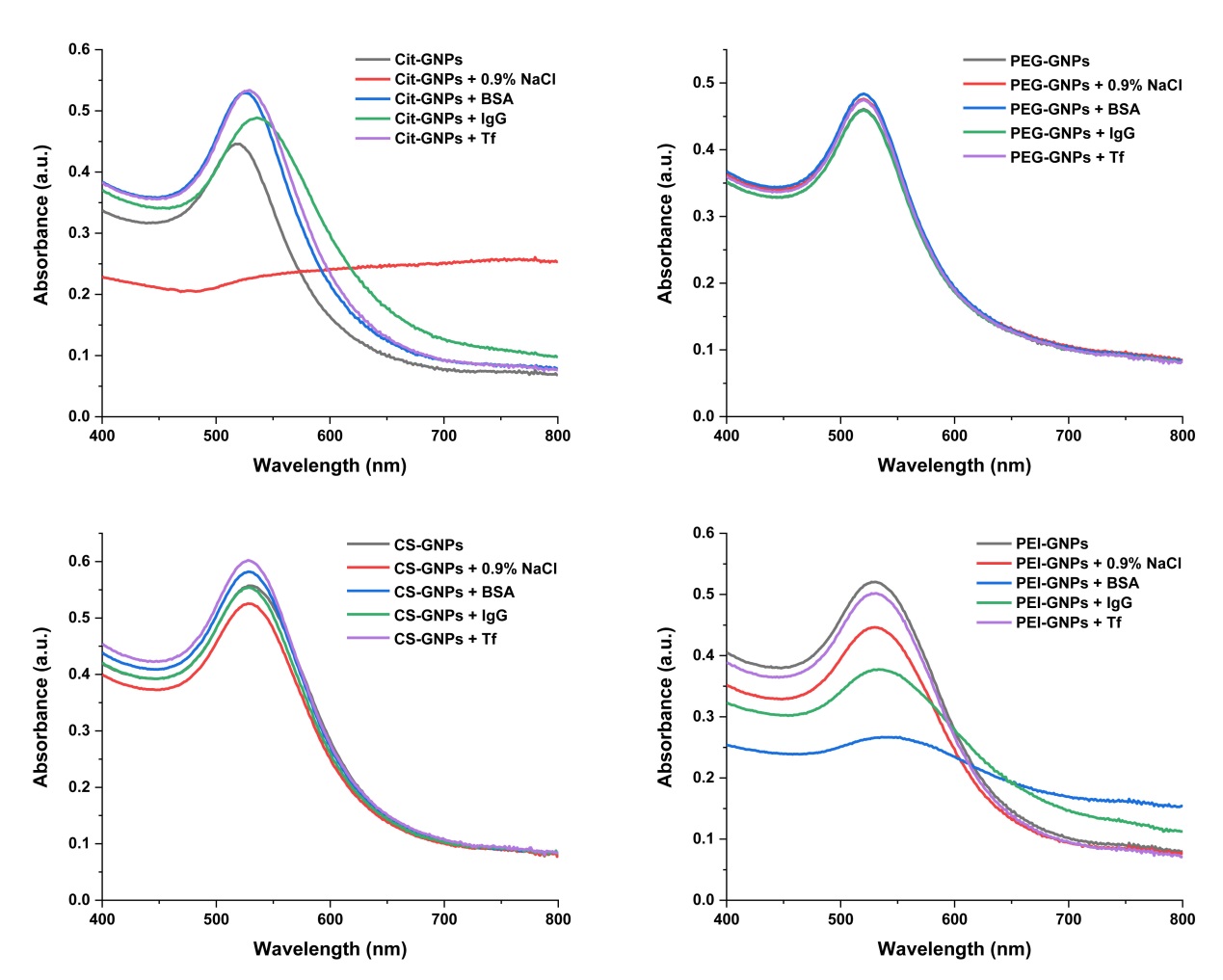
**

Figure S4. UV-Vis spectra of functionalized GNPs after incubation with BSA, IgG and TF solution.

**
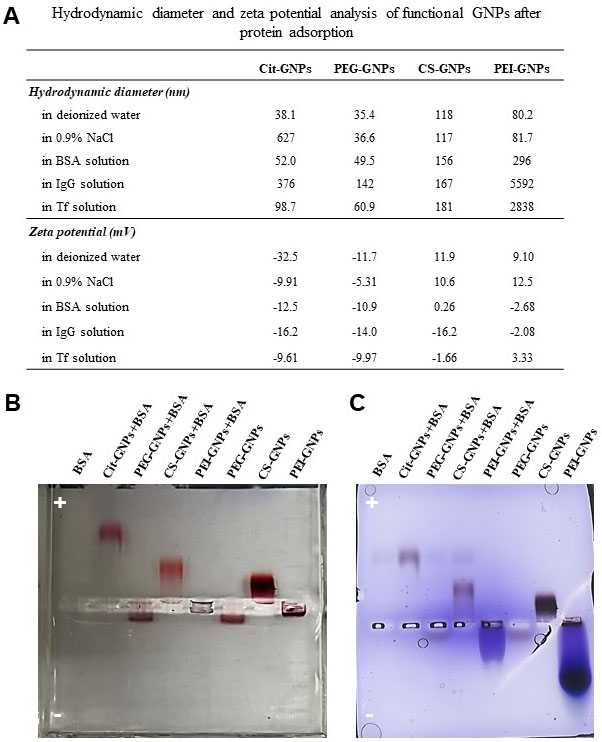
**

Figure S5. Hydrodynamic diameter, zeta potential and agarose gel electrophoresis analysis of PEG-GNPs, CS-GNPs and PEI-GNPs adsorption of serum proteins. (A) Hydrodynamic diameter and zeta potential analysis of GNPs after incubation with proteins; (B) After agarose gel electrophoresis; (C) Coomassie blue staining of protein gel.


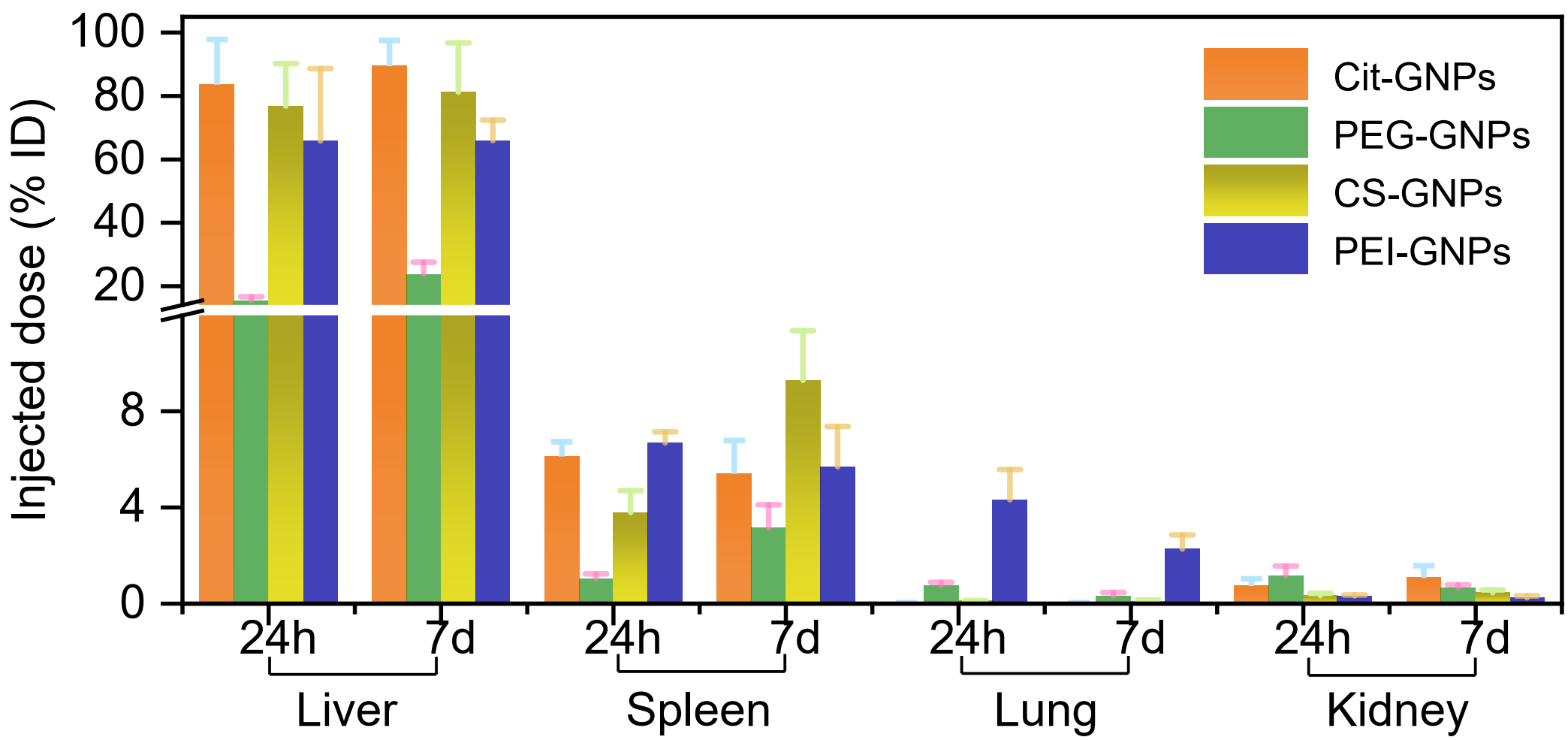


Figure S6. Bioaccumulation of GNPs in the liver, spleen, lung and kidney after injection of Cit-GNPs，PEG-GNPs，PEI-GNPs，CS-GNPs post intravenous for 24h and 7d.


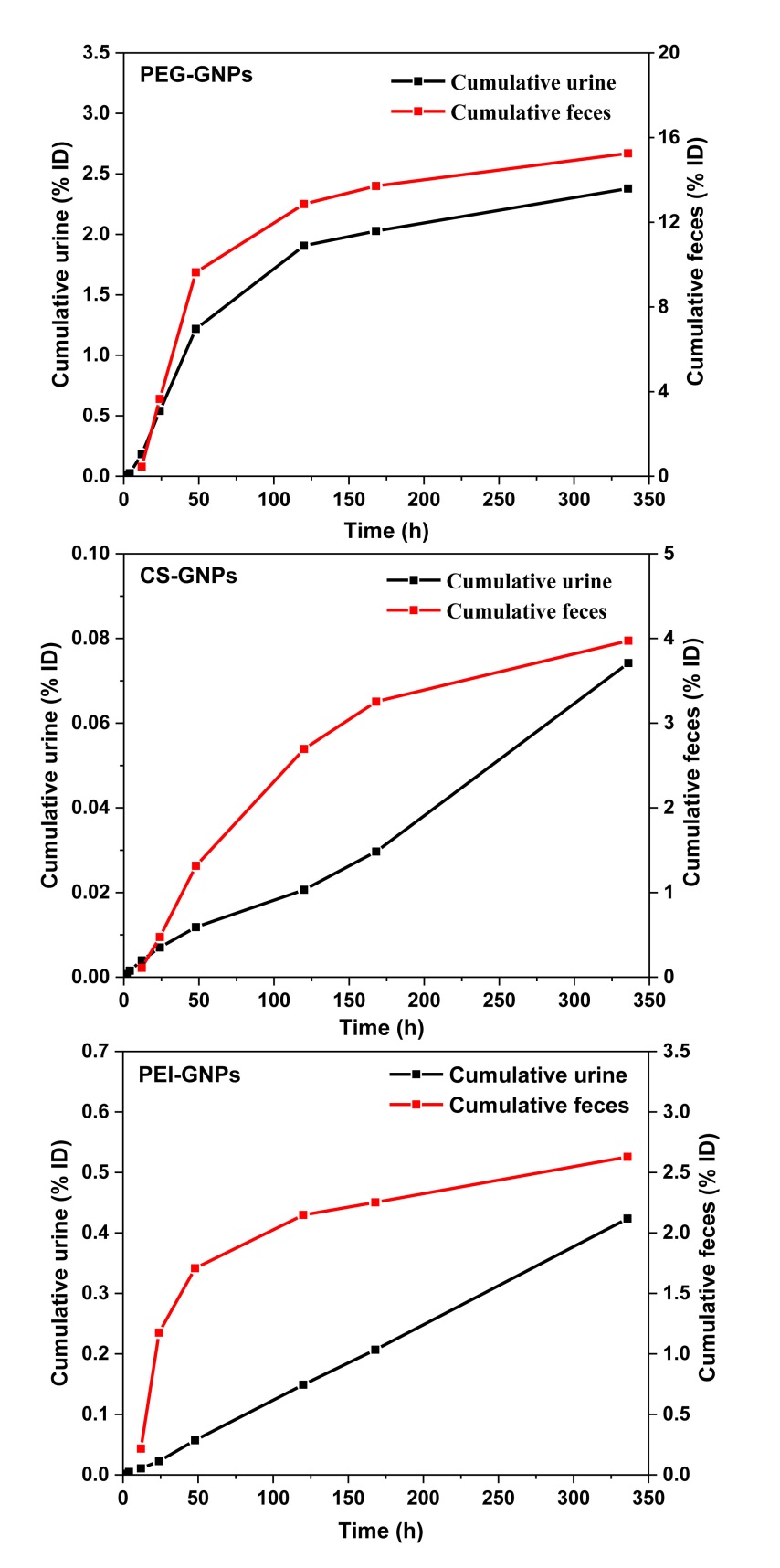


Figure S7. Cumulative amounts of GNPs excreted in urine and feces within 14 days after i.v. injection of PEG-GNPs, CS-GNPs, and PEI-GNPs.

**
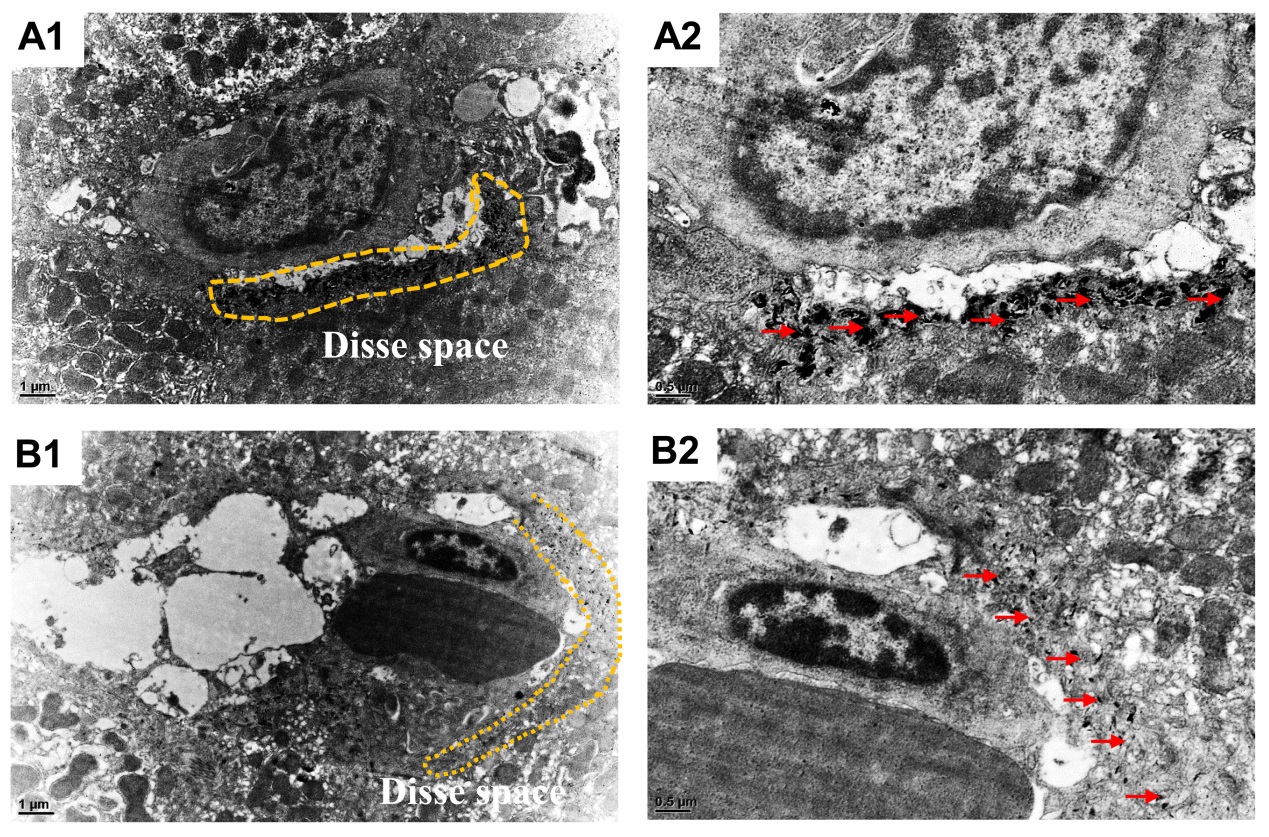
**

Figure S8. PEG-GNPs were observed in Disse space of mouse liver. The right image is a magnified one indicated by the orange dashed line in the left image (scale bar: left, 1 μm; right, 500 nm). Red arrow indicates the deposition of GNPs in the liver.

**
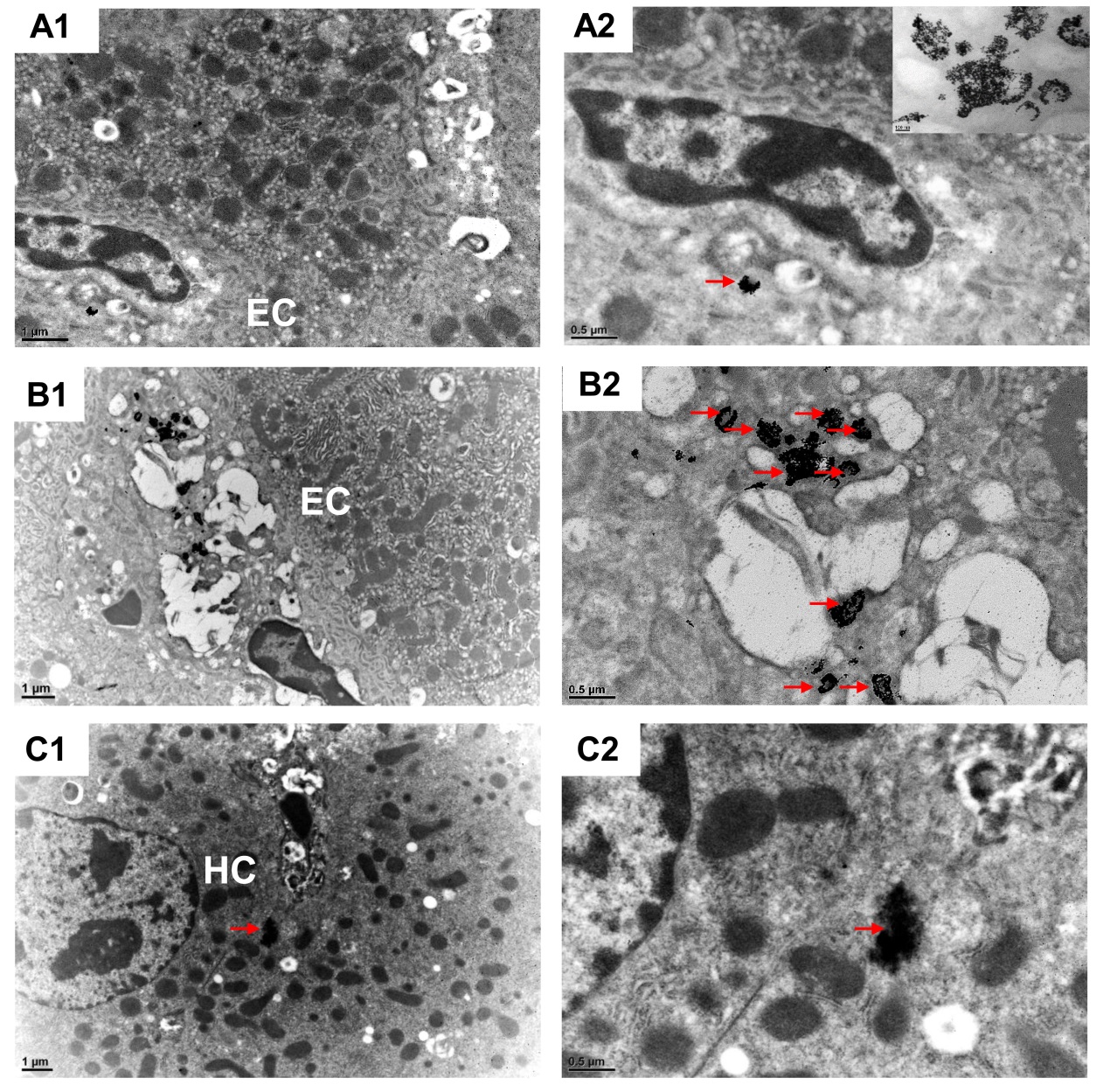
**

Figure S9. Large amount of CS-GNPs were observed in cytosol of endothelial cell (A1-B2) in the liver and induced vacuolar degeneration of endothelial cells (ECs) at 1 h post-injection (B1, B2). Right image is the magnification of the left one. (Scale bar: Left, 1 μm; Right, 500 nm). (C) A little amount of agglomerated CS-GNPs were observed in cytosol of hepatocyte of mouse liver. Red arrow indicates the deposition of GNPs in the liver.

**
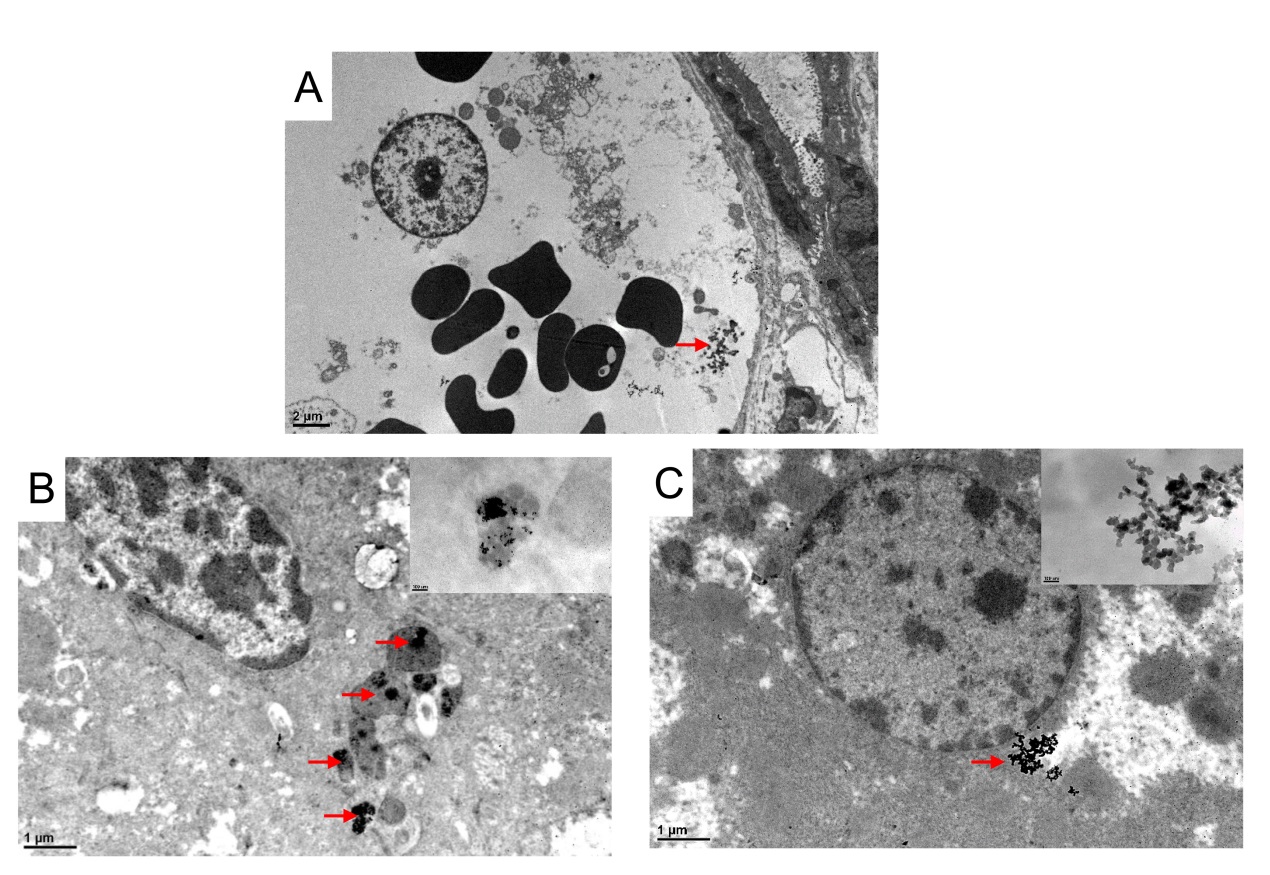
**

Figure S10. TEM image of PEI-GNPs in the mouse liver after i.v. injection. PEI-GNPs were observed in hepatocyte across Disse space (A); in lysosome of KCs (B); and in cytosol of hepatocyte (C) at 1 h post-injection. Red arrow indicates the deposition of GNPs in the liver.

**
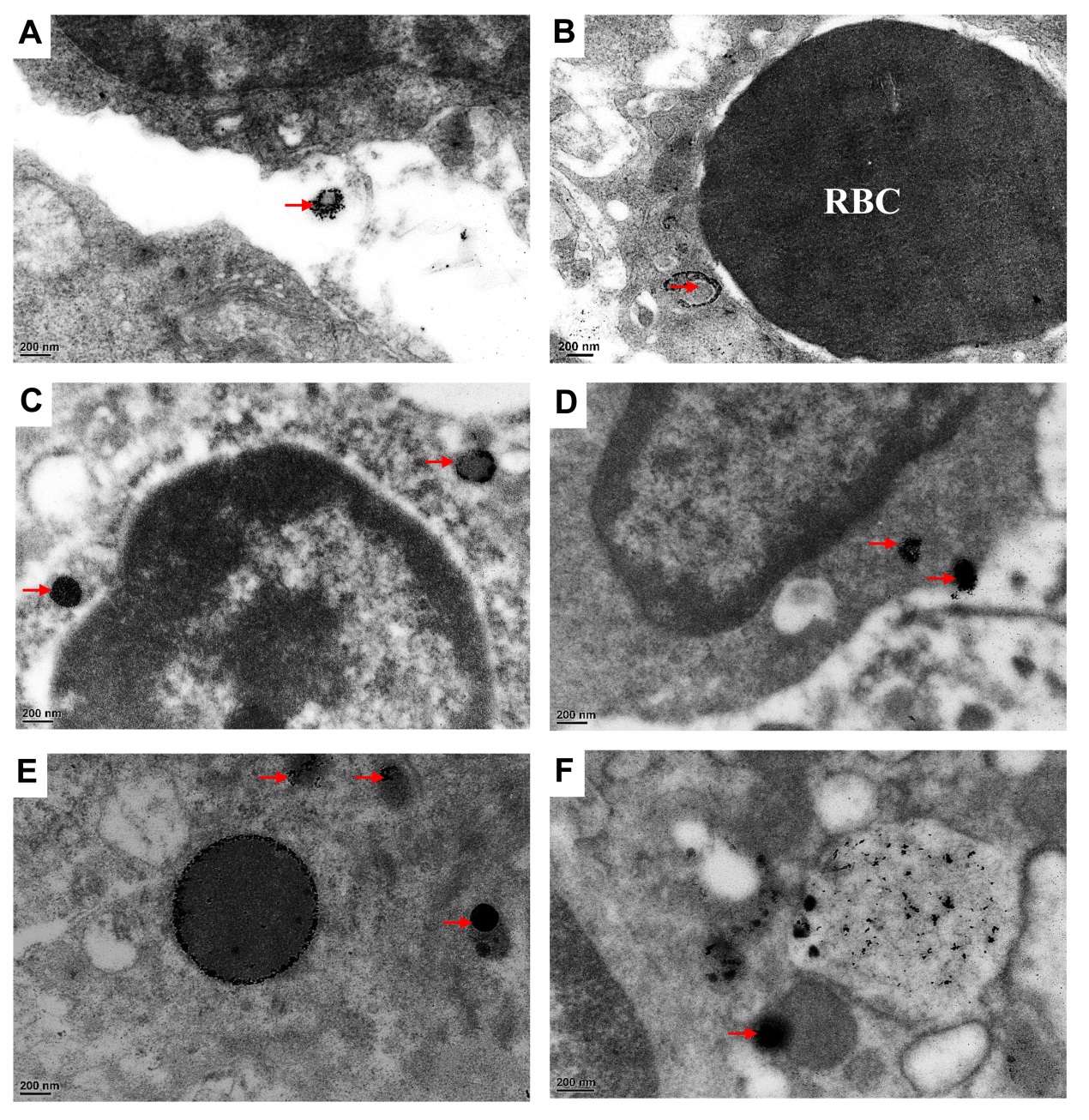
**

Figure S11. TEM image of mouse spleen at 1 h after i.v. injection of PEG-GNPs, CS-GNPs, and PEI-GNPs. (A) PEG-GNPs were deposited in the splenic sinus; (B) PEG-GNPs were phagocytized by macrophage near a red blood cell. CS-GNPs were deposited in the lysosome (C) and cytosol (D) of macrophage. PEI-GNPs were deposited in the lysosome (E) and cytosol (F) of macrophage. Red arrow indicates the deposition of GNPs in the spleen.


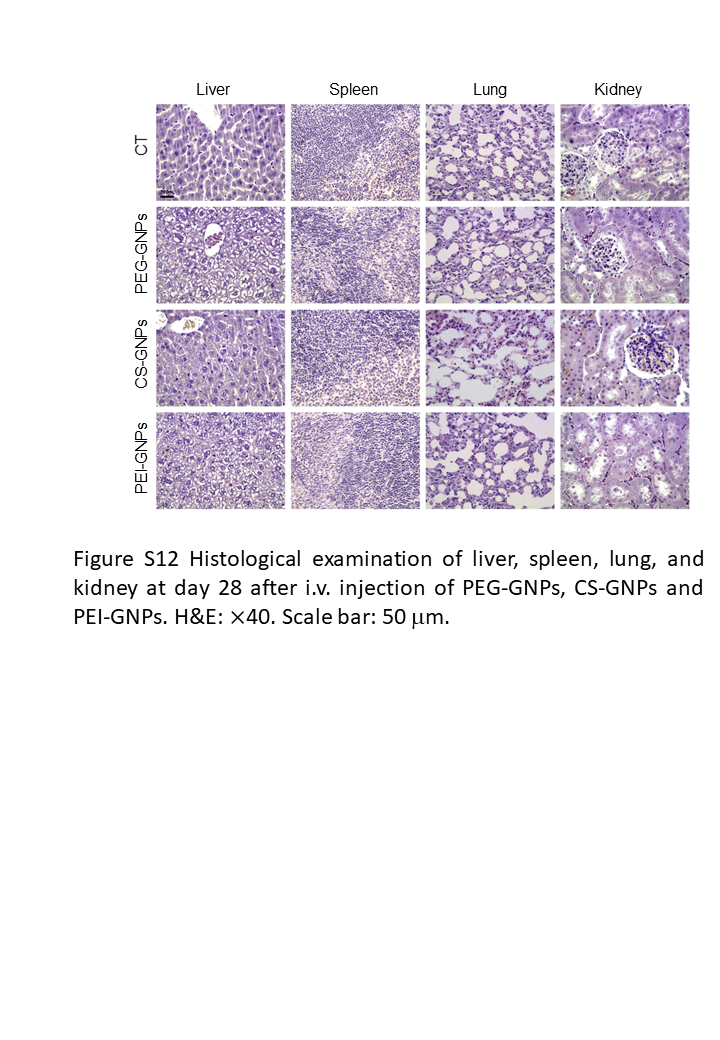


Figure S12. Histological examination of liver, spleen, lung, and kidney at day 28 after i.v. injection of PEG-GNPs, CS-GNPs and PEI-GNPs. H&E: × 40. Scale bar: 50 μm.


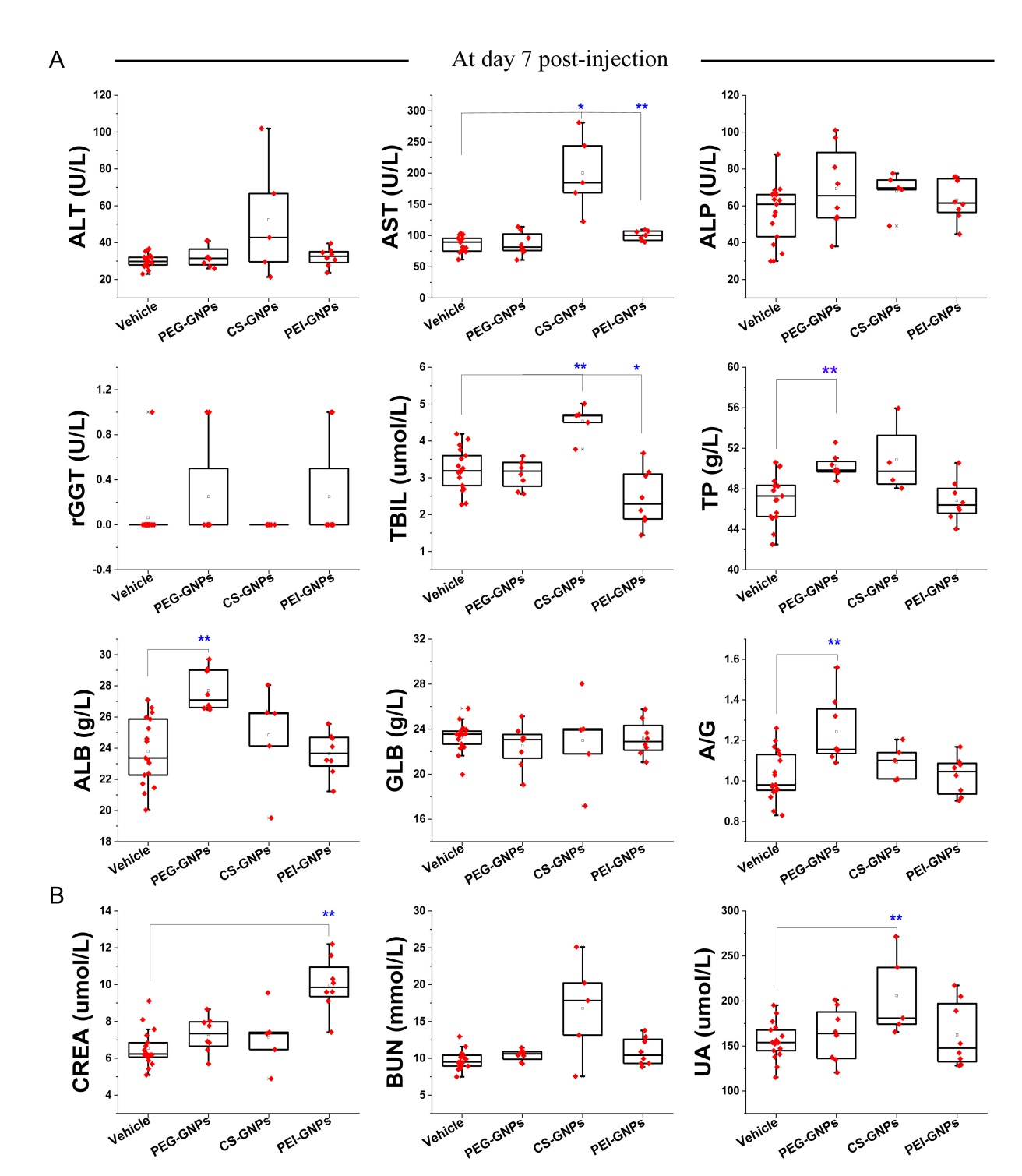


Figure S13. Serum biochemical assay after i.v. injection of PEG-, CS-, PEI-GNPs at day 7. (A) Serum biochemical assay for liver function test; (B) Serum biochemical assay for lidney function test.


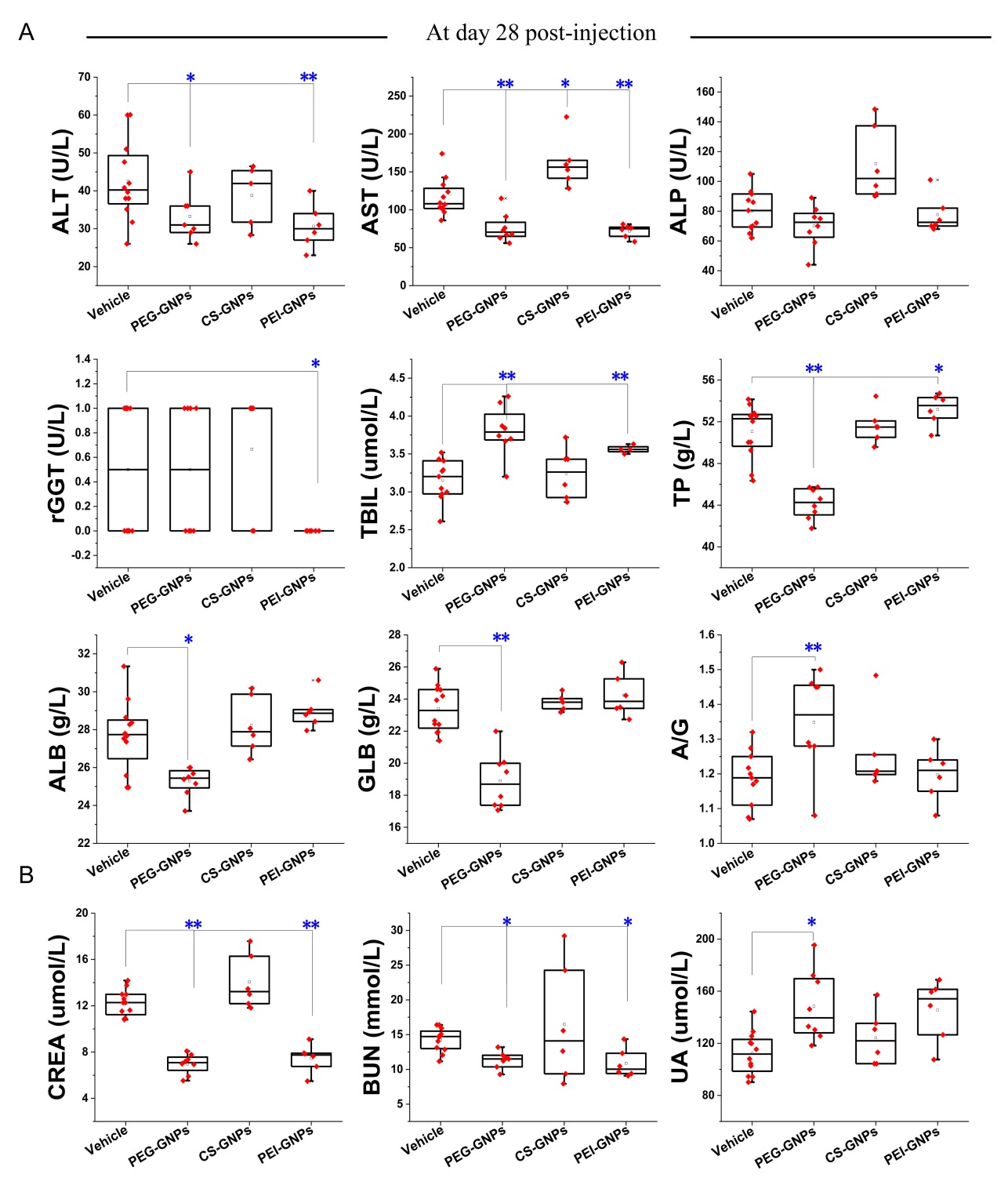


Figure S14. Serum biochemical assay after i.v. injection of PEG-, CS-, PEI-GNPs at day 28. (A) Serum biochemical assay for liver function test; (B) Serum biochemical assay for lidney function test.

**
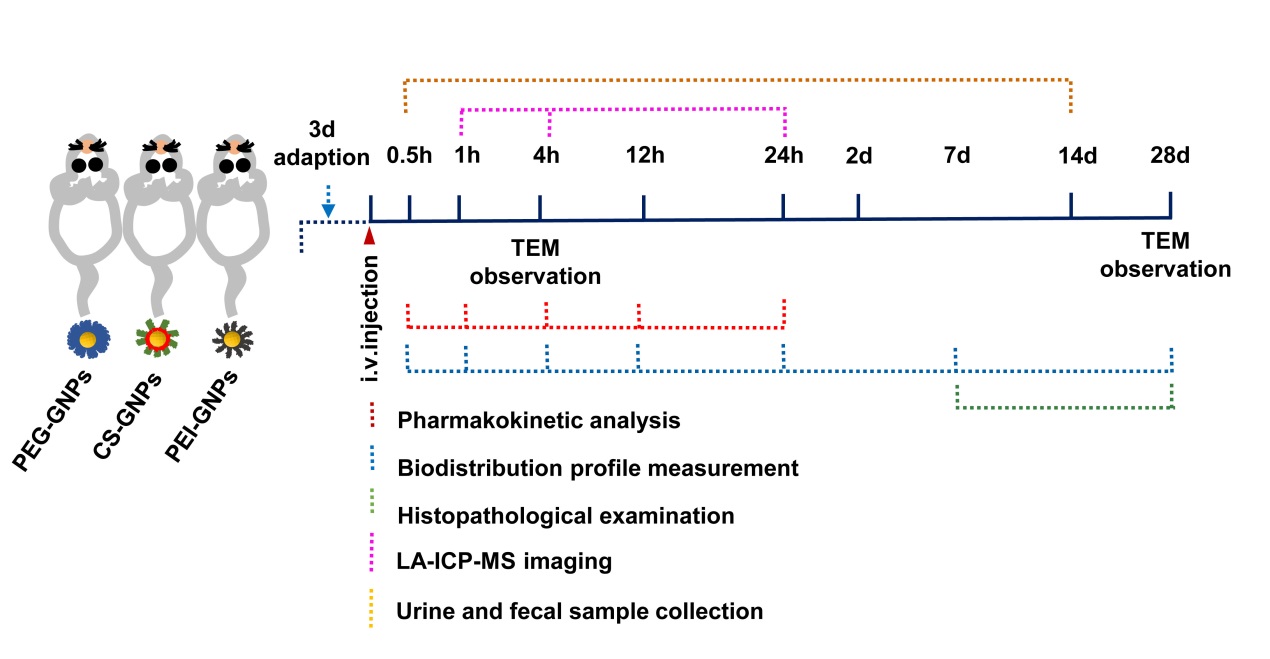
**

Figure S15. Schematic of animal experiments.
